# Advances on strictly $\Delta$-modular IPs

**DOI:** 10.1007/s10107-024-02148-2

**Published:** 2024-10-30

**Authors:** Martin Nägele, Christian Nöbel, Richard Santiago, Rico Zenklusen

**Affiliations:** Department of Mathematics ETH Zurich Raemistrasse 101, Zurich, 8092 Switzerland

**Keywords:** Bounded subdeterminants, Congruency constraints, Integer programming, Group constraints, Total unimodularity, 90C10 Integer programming, 90C27 Combinatorial optimization, 68Q25 Analysis of algorithms and problem complexity

## Abstract

There has been significant work recently on integer programs (IPs) $\min \{c^\top x :Ax\le b,\,x\in \mathbb {Z}^n\}$ with a constraint marix *A* with bounded subdeterminants. This is motivated by a well-known conjecture claiming that, for any constant $\Delta \in \mathbb {Z}_{>0}$, $\Delta$-modular IPs are efficiently solvable, which are IPs where the constraint matrix $A\in \mathbb {Z}^{m\times n}$ has full column rank and all $n\times n$ minors of *A* are within $\{-\Delta , \dots , \Delta \}$. Previous progress on this question, in particular for $\Delta =2$, relies on algorithms that solve an important special case, namely *strictly* $\Delta$-*modular IPs*, which further restrict the $n\times n$ minors of *A* to be within $\{-\Delta , 0, \Delta \}$. Even for $\Delta =2$, such problems include well-known combinatorial optimization problems like the minimum odd/even cut problem. The conjecture remains open even for strictly $\Delta$-modular IPs. Prior advances were restricted to prime $\Delta$, which allows for employing strong number-theoretic results. In this work, we make first progress beyond the prime case by presenting techniques not relying on such strong number-theoretic prime results. In particular, our approach implies that there is a randomized algorithm to check feasibility of strictly $\Delta$-modular IPs in strongly polynomial time if $\Delta \le 4$.

## Introduction

Integer Programs (IPs) $\min \{c^\top x:Ax\le b,\, x\in \mathbb {Z}^n\}$ are a central NP-hard problem class in Combinatorial Optimization. There is substantial prior work and interest in identifying special classes of polynomial-time solvable IPs while remaining as general as possible. One of the best-known such classes are IPs with a constraint matrix that is *totally unimodular* (TU), i.e., the determinant of any of its square submatrices is within $\{-1,0,1\}$. A long-standing open conjecture in the field is whether this result can be generalized to $\Delta$-modular constraint matrices for constant $\Delta$. Here, we say that a matrix $A\in \mathbb {Z}^{k\times n}$ is $\Delta$-*modular* if it has full column rank and all $n\times n$ submatrices have determinants in $\{-\Delta ,\ldots ,\Delta \}$.^1^ For brevity, we call an IP with $\Delta$-modular constraint matrix a $\Delta$-*modular IP*. We recap the above-mentioned conjecture below. Unfortunately, we do not know its precise origin; it may be considered folklore in the field.

### Conjecture 1

For constant $\Delta \in \mathbb {Z}_{\ge 0}$, $\Delta$-modular IPs can be solved in polynomial time.

First progress on Conjecture 1 was made by [1], who showed that it holds for $\Delta =2$ (the bimodular case). [2] show that the conjecture is true for an arbitrary constant $\Delta$ under the extra condition that the constraint matrix has at most two non-zero entries per row or column. Through a non-trivial extension of the techniques in [1], it was shown by [3] that there is a randomized algorithm to check feasibility of an IP with a strictly 3-modular constraint matrix in polynomial time. Here, a matrix $A\in \mathbb {Z}^{k\times n}$ is called *strictly* $\Delta$-*modular* if it has full column rank and all its $n\times n$ submatrices have determinants in $\{-\Delta , 0, \Delta \}$.

As a key ingredient, all these prior approaches solve certain combinatorial optimization problems with congruency constraints. This is not surprising, as even strictly $\Delta$-modular IPs include the following class of *MCCTU problems*^2^
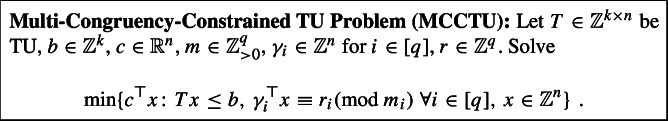
 Unless mentioned otherwise, we assume that in the context of MCCTU problems, *q* and all $m_i$’s are constant. Even MCCTU with just a single congruency constraint, i.e., $q=1$, already contains the classical and well-studied odd and even cut problems, and, more generally, the problem of finding a minimum cut whose number of vertices is $r \, (\text{ mod } m)$. (See [4–9] for related work.) It can also capture the minimum *T*-join problem, congruency-constrained flow problems, and many other problems linked to TU matrices.

Combinatorial optimization problems with congruency constraints are highly non-trivial and many open questions remain. As they are already captured by strictly $\Delta$-modular IPs, this motivates the following weakening of Conjecture 1.

### Conjecture 2

Strictly $\Delta$-modular IPs can be solved in polynomial time for constant $\Delta \in \mathbb {Z}_{\ge 0}$.

Even resolving this weaker conjecture would settle several open problems, including congruency-constrained min cuts (in both directed and undirected graphs), or the problem of efficiently and deterministically finding a perfect matching in a red/blue edge-colored bipartite graph such that the number of red matching edges is $r \, (\text{ mod } m)$. (This is a simplified version of the famous red-blue matching problem, where the task is to find a perfect matching with a specified number of red edges; for both versions, randomized algorithms are known.) Interestingly, for the bimodular case ($\Delta =2$), a result by [10] implies that Conjecture 1 and Conjecture 2 are equivalent (see [1]).

Our goal is to shed further light on Conjecture 2 and overcome some important hurdles of prior approaches. In a first step, we note that a positive resolution of Conjecture 2 does not only imply efficient solvability of MCCTU problems, but also vice versa, and this backward reduction works in strongly polynomial time.

### Lemma 3

Let $\Delta >0$. Every strictly $\Delta$-modular IP can, in strongly polynomial time, be reduced to an MCCTU problem with moduli $m_i$ such that $\Delta =\prod _{i=1}^q m_i$.

Without the strongly polynomial time condition, this also follows from very recent work of [11, Lemma 4].

Further, we are interested in making progress regarding the feasibility version of Conjecture 2, i.e., efficiently deciding whether a strictly $\Delta$-modular IP is feasible. Prior approaches settle this question for $\Delta =2$ [1] and—using a randomized algorithm— for $\Delta =3$ [3]. A main hurdle to extend these is that they crucially rely on $\Delta$ being prime, for example through the use of the Cauchy-Davenport Theorem. Our main contribution here is to address this. In particular, we can check feasibility for $\Delta =4$ with a randomized algorithm, which is the first result in this context for non-prime $\Delta$. More importantly, our techniques will hopefully prove useful for future advances on this challenging question.

### Theorem 4

There exists a strongly polynomial-time randomized algorithm to find a feasible solution of a strictly 4-modular IP, or detect that it is infeasible.

We remark that the randomization appearing in the above theorem comes from the fact that one building block of our result is a reduction to a problem class that includes the aforementioned congruency-constrained red/blue-perfect matching problem, for which only randomized approaches are known (except for restricted graph classes [12, 13]).

### Group-constrained problems and proof strategy for Theorem 4

To show Theorem 4, we exploit its close connection to MCCTU. Capturing the congruency constraints of an MCCTU problem through an abelian group constraint, we attain the following *group-constrained TU feasibility problem*. 
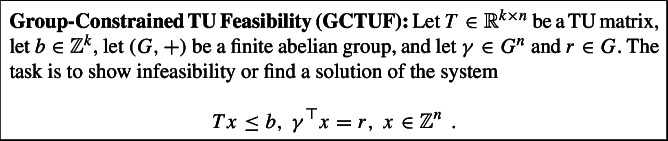
 Here, the scalar product $\gamma ^\top x$ denotes the linear combination of the group elements $\gamma _1,\ldots ,\gamma _n$ with multiplicities $x_1,\ldots ,x_n$ in *G*. Group constraints generalize single congruency constraints, which are obtained in the special case where *G* is cyclic. More generally, by the fundamental theorem of finite abelian groups, a finite abelian group *G* is, up to isomorphism, a direct product of cyclic groups. Hence, a group constraint can be interpreted as a set of congruency constraints and vice versa. Thus, GCTUF and MCCTU feasibility are two views on the same problem. We stick to GCTUF mostly for convenience of notation. Moreover, the GCTUF setting also allows for an elegant use of group-related results later on. One may assume that the group is given through its multiplication table (the *Cayley table*). In fact, the precise group representation is not of great importance to us. Concretely, strictly $\Delta$-modular IP feasibility problems reduce to GCTUF problems with a group of size $\Delta$, and $\Delta$ is typically assumed to be a constant. Many of our polynomial-time algorithmic results can even be extended to settings where the group size is not part of the input, and access to group operations is provided through an oracle.

By a slight extension of Lemma 3 (see Sect. 2) and the aforementioned equivalent viewpoint of multiple congruency constraints and a group constraint, in order to prove Theorem 4, it is enough for us to show the equivalent statement below.

#### Theorem 5

There exists a strongly polynomial time randomized algorithm for GCTUF problems with a group of cardinality at most 4.

On a high level, we follow a well-known strategy for TU-related problems by employing Seymour’s decomposition [14] to decompose the problem into problems on simpler, more structured TU matrices. (See, e.g., [1, 3, 15, 16].) Roughly speaking, Seymour’s decomposition states that a TU matrix is either very structured—in which case we call it a *base block*—or can be decomposed into smaller TU matrices through a small set of well-defined operations. (See Theorem 15 and the disucssion thereafter for more details and a formal definition of *base blocks*.) The use of Seymour’s decomposition typically comes with two main challenges, namely

1. Solving the base block cases, and
2. Propagating solutions of the base block cases back through the decomposition efficiently to solve the original problem.

First, we show that this propagation can be done efficiently for our problem.

#### Theorem 6

Let *G* be an abelian group of size at most 4. Given an oracle for solving base block GCTUF problems with group *G*, we can solve GCTUF problems with group *G* in strongly polynomial time with strongly polynomially many calls to the oracle.

In fact, our approach underlying Theorems 5 and 6 operates in a hierarchy of GCTUF problems with increasingly relaxed group constraints of the form $\gamma ^\top x\in R$ for subsets $R\subseteq G$ of increasing size, and allows for proving the above results for such relaxed GCTUF problems for arbitrary constant-size groups *G* as long as $|G|-|R|\le 3$. (See Sect. 4 for more details.) In principle, this is along the lines of the approach to congruency-constrained TU problems in [3], but incorporates the new viewpoint of group constraints, and additionally improves over earlier results in two ways: First, our approach applies to arbitrary finite abelian groups, while previous setups heavily relied on the group cardinality being a prime. Secondly, in the setting with relaxed group constraints, we extend the admissible range of $|G|-|R|$ by one, thus proceeding further in the hierarchy of GCTUF problems, and newly covering GCTUF problems with groups of cardinality 4.

Besides being a key part of our approach, Theorem 6 underlines that base block GCTUF problems are not merely special cases, but play a key role in progress on general GCTUF problems. There are only two non-trivial types of such base block GCTUF problems, namely when the constraint matrix is a so-called *network matrix* or a transpose thereof. Both cases cover combinatorial problems that are interesting on their own, and their complexity status remains open to date. If the constraint matrix is a network matrix, GCTUF can be cast as a circulation problem with a group constraint. By reducing to and exploiting results of [17] on exact perfect matching problems, a randomized algorithm for the congruency-constrained case has been presented in [3]. We observe that these results extend to the group-constrained setting. The other base block case, where the constraint matrix is the transpose of a network matrix, can be cast as a group-constrained directed minimum cut problem by leveraging a result in [3]. Prior work combined this reduction with results on congruency-constrained submodular minimization [8] to solve the optimization version of the problem for congruency-constraints of prime power modulus. We show that the feasibility question on this base block can be solved efficiently on any finite abelian group of constant order, thus circumventing the prime power restriction that is intrinsic in prior approaches.

#### Theorem 7

Let *G* be a finite abelian group. There is a strongly polynomial time algorithm for solving GCTUF problems with group *G* where the constraint matrix is the transpose of a network matrix.

### Further related work

The parameter $\Delta$ has been studied from various viewpoints. While efficient recognition of (totally) $\Delta$-modular matrices is open for any $\Delta \ge 2$, approaches to approximate the largest subdeterminant in absolute value were studied [18, 19]. Also, focusing on more restricted subdeterminant patterns proved useful [10, 20, 21]. Aiming at generalizing a bound of [22] for $\Delta =1$, bounds on the maximum number of rows of a $\Delta$-modular matrix were obtained [23–25]. Also, the influence of the parameter $\Delta$ on structure and properties of IPs and polyhedra is multi-faceted (see, e.g., [26–33] and references therein).

### Structure of the paper

We prove the strongly polynomial time reduction from Lemma 3 in Sect. 2. In Sect. 3, we prove Theorem 7. Section 4 illustrates our approach and new contributions towards Theorem 6 on a more technical level, explains the main new ingredients of our proof, and discusses a main roadblock when trying to directly extend our approach to $\Delta > 4$.

## Reducing to group-constrained problems

We prove the following slightly strengthened version of Lemma 3.

### Lemma 8

Let $\Delta >0$. Given a strictly $\Delta$-modular IP of the form $\min \{c^\top x:Ax\le b, x\in \mathbb {Z}^n\}$, one can, in strongly polynomial time, determine an MCCTU problem

$$\begin{aligned} \min \{\bar{c}^\top y :Ty \le b,\, \gamma _i^\top y \equiv r_i \, (\text{ mod } m_i) \;\forall i\in [q],\, y\in \mathbb {Z}^n\} \end{aligned}$$

together with a non-singular $n\times n$ submatrix *H* of *A* such that the following holds:

(i) $$\Delta =\prod _{i\in [q]}m_i$$.
(ii) $$\bar{c}^\top =c^\top H^{-1}$$.
(iii) The map $x\mapsto Hx$ is a bijection between feasible solutions of the strictly $\Delta$-modular IP and the MCCTU problem.

We remark that the one-to-one correspondence of feasible solutions given in Item (iii) of Lemma 8 is (besides Item (i)) precisely what we need to deduce our main result, Theorem 4, from Theorem 5. Moreover, Items (ii) and (iii) of Lemma 8 together imply that $x\mapsto Hx$ is not only a bijection between feasible solutions, but also a bijection between optimal solutions of the two involved problems, so Lemma 3 is indeed also implied by Lemma 8.

### Proof of Lemma 8

We show how to transform the given strictly $\Delta$-modular problem into an MCCTU problem. Let *H* be an $n\times n$ submatrix of *A* with $|\det (H)|=\Delta$. After a variable transformation to $y=Hx$, we can equivalently rewrite the original integer program in the form

$$ \min \{\bar{c}^\top y:Ty \le b,\, H^{-1}y\in \mathbb {Z}^n\}\hspace{5.0pt}, $$

where $\bar{c}^\top =c^\top H^{-1}$, and $T=AH^{-1}$. By definition, the map $x\mapsto Hx$ is a bijection between feasible solutions of the original IP and the above problem. Note that *T* is unimodular and contains an identity submatrix; hence *T* is totally unimodular. To complete the proof, it thus suffices to show that the constraint $H^{-1}y\in \mathbb {Z}^n$ can be transformed to multiple congruency constraints with moduli whose product equals $\Delta$.

To this end, we first write $H^{-1} = H_I + H_F$ with an integer matrix $H_I\in \mathbb {Z}^{n\times n}$ and a fractional matrix $H_F\in [0,1)^{n\times n}$, i.e., matrices whose entries are given by

$$ (H_I)_{i,j} :==\left\lfloor \left( H^{-1}\right) _{i,j} \right\rfloor \quad \text {and}\quad (H_F)_{i,j} :==\left( H^{-1}\right) _{i,j} - (H_I)_{i,j}\hspace{5.0pt}, $$

where, for $x\in \mathbb {R}$, $\lfloor x\rfloor$ is the integer part of *x*, i.e., the unique number $n\in \mathbb {Z}$ with $n\le x<n+1$. Using this decomposition, we obtain

$$ H^{-1}y\in \mathbb {Z}^n \quad \iff \quad H_Iy + H_Fy\in \mathbb {Z}^n \quad \iff \quad H_Fy\in \mathbb {Z}^n\hspace{5.0pt}. $$

Because $\det (H)=\Delta$, we have $\Delta H^{-1}\in \mathbb {Z}^{n\times n}$ by Cramer’s rule, and thus also $\widetilde{H}_F:==\Delta H_F\in \mathbb {Z}^{n\times n}$. Furthermore, the entries of $\widetilde{H}_F$ are bounded by the constant $\Delta$ in absolute value, thus any weakly polynomial time algorithm applied to $\widetilde{H}_F$ even runs in strongly polynonmial time. Consequently, using a weakly polynomial time algorithm for computing the Smith normal form of an integer matrix [34], we can in strongly polynomial time determine the Smith normal form of $\widetilde{H}_F$, i.e., we can in strongly polynomial time find unimodular matrices $S, U\in \mathbb {Z}^{n\times n}$ and integers $\widetilde{m}_i\in \mathbb {Z}$ such that $D={\text {diag}}(\widetilde{m}_1, \ldots , \widetilde{m}_n)=S^{-1}\widetilde{H}_F U^{-1}$. Using this decomposition, we get

$$ H_Fy\in \mathbb {Z}^n \quad \iff \quad SDUy\in \Delta \mathbb {Z}^n \quad \iff \quad DUy\in \Delta \mathbb {Z}^n\hspace{5.0pt}. $$

Here, the last equivalence exploits unimodularity of *S*. Letting $\gamma _i^\top$ denote the *i*^th^ row of *U*, we can further rewrite

$$\begin{aligned} DUy\in \Delta \mathbb {Z}^n {\quad }{\;}\iff {\quad }{\;} \forall i\in [n]:\ \widetilde{m}_i \gamma _i^\top y \in \Delta \mathbb {Z}^n {\quad }{\;}\iff {\quad }{\;} \forall i\in [n]:\ \gamma _i^\top y \equiv 0\, (\text{ mod } {m_i}), \end{aligned}$$

where we use $m_i:==\Delta /\gcd (\Delta , \widetilde{m}_i)$.^3^ It is thus left to show $\Delta =\prod _{i=1}^n m_i$. To this end, consider the composed map

$$ \Phi :\mathbb {Z}^n \overset{U}{\longrightarrow }\ \mathbb {Z}^n \overset{\pi }{\longrightarrow }\ \prod _{i=1}^n \nicefrac {\mathbb {Z}}{m_i\mathbb {Z}}\hspace{5.0pt}, $$

where, for $z\in \mathbb {Z}^n$, the first map is defined by $z\mapsto Uz$, and the second is the component-wise projection given by $\pi (z):==(\pi _1(z_1),\ldots ,\pi _n(z_n))$, where $\pi _i$ denotes the natural projection from $\mathbb {Z}$ to $\mathbb {Z}/m_i\mathbb {Z}$. As *U* is unimodular, the first map is an isomorphism of groups. Furthermore, $\pi$ is a surjective group homomorphism. By the isomorphism theorem, we thus get an isomorphism

$$ \prod _{i=1}^n \nicefrac {\mathbb {Z}}{m_i\mathbb {Z}} = {\text {im}}\Phi \cong \nicefrac {\mathbb {Z}^n}{\ker \Phi }\hspace{5.0pt}. $$

Note that the cardinality of the left-hand side group is $\prod _{i=1}^n m_i$. Therefore, we may finish the proof by showing that $|\mathbb {Z}^n/\ker \Phi |= \Delta$. To this end, observe that $\ker \Phi$ is the set of $y\in \mathbb {Z}^n$ fulfilling the congruency constraints, i.e., $\gamma _i^\top y\equiv 0 \, (\text{ mod } m_i)$ for all $i\in [n]$. By the above discussion, this is precisely the set $\{y \in \mathbb {Z}^n:H^{-1}y\in \mathbb {Z}^n\} = H\mathbb {Z}^n$. Consequently, $|\mathbb {Z}^n/\ker \Phi |= |\mathbb {Z}^n/H\mathbb {Z}^n|=|\det (H)|=\Delta$, as desired. $\square$

The proof of Lemma 8 implies a few interesting properties of strictly $\Delta$-modular matrices. First, every $\Delta$-modular matrix $A\in \mathbb {Z}^{k\times n}$ can be written as $A=TH$ for a square matrix $H\in \mathbb {Z}^{n\times n}$ with $\det (H)=\Delta$ and a totally unimodular matrix $T\in \mathbb {Z}^{k\times n}$. In particular, the number of distinct rows of *A* is equal to the number of distinct rows of *T*. By a result of [22], the number of distinct rows of *T* is bounded by $n^2+n+1$, so the same bound holds for strictly $\Delta$-modular matrices.

Furthermore, it turns out that the existence of this decomposition is equivalent to strict $\Delta$-modularity. This allows us to detect strict $\Delta$-modularity in polynomial time by showing that one can find such a decomposition in polynomial time. We can efficiently check whether *A* has full column rank and, if it does, identify an $n\times n$ submatrix *H* of full rank. If $\det (H)\notin \{-\Delta , \Delta \}$, *A* is not strictly $\Delta$-modular. Else, consider $T=H^{-1}A$. The proof of Lemma 8 shows that *T* is totally unimodular if *A* is strictly $\Delta$-modular. Vice versa, assume that *T* is totally unimodular. Then every $n\times n$ submatrix of *A* can be written as an $n\times n$ submatrix of *T* times the matrix *H*. So in particular it has a determinant in $\{-\Delta , 0, \Delta \}$. Hence *A* is strictly $\Delta$-modular. Altogether, *A* is strictly $\Delta$-modular if and only if *T* is totally unimodular. The latter can be determined using a polynomial time test for total unimodularity [35].

## GCTUF with transposed network constraint matrices

In the setting with a congruency constraint instead of a group constraint, [3] shows that every base block problem with a constraint matrix that is a transposed network matrix can be reduced to a node-weighted minimization problem over a lattice with a congruency constraint,^4^ i.e., a problem of the form

1 $$\begin{aligned} \min \{w(S):S\in \mathcal {L},\,\gamma (S)\equiv r\, (\text{ mod } m) \}\hspace{5.0pt}, \end{aligned}$$

where $\mathcal {L}\subseteq 2^N$ is a lattice on some finite ground set *N*, $\gamma :N\rightarrow \mathbb {Z}$, $r\in \mathbb {Z}$, $m\in \mathbb {Z}_{>0}$, $w:N\rightarrow \mathbb {R}$, and we use $\gamma (S):==\sum _{v\in S}\gamma (v)$ as well as $w(S):==\sum _{v\in S}w(v)$.^5^ Being a special case of congruency-constrained submodular minimization, it is known that such problems, and thus the corresponding congruency-constrained TU problems with a transposed network constraint matrix, can be solved in strongly polynomial time for constant prime power moduli *m*, while the case of general constant composite moduli remains open [8]. The progress on GCTUF , particularly the reduction to base block feasibility problems through Theorem 6 and its generalization (Theorem 16 in Sect. 4), motivates studying these reductions and results in the feasibility setting and with a group constraint instead of a congruency constraint, giving rise to the following problem.
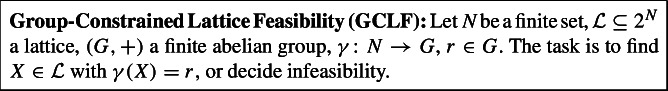


In [3, Section 4.2], the following result was shown implicitly for the case of a cyclic group *G*.

### Theorem 9

Given a finite abelian group *G*, consider a GCTUF problem on *n* variables and a constraint matrix that is the transpose of a network matrix. One can in strongly polynomial time determine a GCLF problem over a ground set *N* with $|N| = n|G|$ such that from a solution of the GCLF problem, we can in strongly polynomial time compute a solution of the GCTUF problem.

The statement was shown in the more general setting of optimization, but in our context the special case of feasiblity suffices. It turns out, that no properties of cyclic groups beyond them being finite abelian groups are used in the proofs, hence by substituting all occurrences of congruency constraints, i.e., constraints in a cyclic group $\mathbb {Z}/m\mathbb {Z}$, with group constraints, the original proofs also imply the theorem for a general finite abelian group. Thus, it remains to study GCLF problems. Interestingly, for the pure feasibility question, we can circumvent the barriers present in the optimization setting, and obtain the following result through a concise argument.

### Theorem 10

Let *G* be a finite abelian group. GCLF problems with group *G* can be solved in strongly polynomial time.

Clearly, Theorem 9 and Theorem 10 together imply Theorem 7. The main observation towards a proof of Theorem 10 is the following elementary lemma.

### Lemma 11

Let *G* be a finite abelian group, and let $\gamma _1,\ldots ,\gamma _\ell \in G$. If $\ell \ge |G|$, then there is a non-empty subset $I\subseteq [\ell ]$ such that $\sum _{i\in I} \gamma _i=0$.

### Proof

Either $s_i:==\sum _{j\le i}\gamma _j=0$ for some $i\in [\ell ]$, or there exist $i<j$ with $s_i=s_j$; hence $I=[i]$ or $I=\{i+1,\ldots ,j\}$, respectively, has the desired properties. $\square$

To prove Theorem 10, we work with a representation of the lattice $\mathcal {L}$ through an acyclic digraph *H* (see Footnote 5). We exploit that every $X\in \mathcal {L}$ is uniquely defined by the subset $C_X:==\{x\in X:\delta ^+(x)\subseteq \delta ^+(X)\}$.

### Proof of Theorem 10

We claim that if the given GCLF problem is feasible, there is a feasible *X* with $|C_X| < |G|$. If so, we obtain an efficient procedure for GCLF with group *G* through enumerating all such $C_X$ and checking if $\gamma (X)=r$. To prove the claim, assume for contradiction that it is wrong, and let $X\in \mathcal {L}$ be minimal with $\gamma (X) = r$. Then $|C_X| \ge |G|$, and applying Lemma 11 to $C_X$ gives a non-empty subset $Y\subseteq C_X$ with $\gamma (Y) = 0$. Thus, $X{\setminus } Y$ is a strictly smaller lattice element with $\gamma (X{\setminus } Y) = \gamma (X) - \gamma (Y) = \gamma (X) = r$, a contradiction.

## Approaching GCTUF problems and a proof of Theorem 6

In order to tackle GCTUF problems, following ideas from [3], we introduce a hierarchy of slightly relaxed GCTUF problems by weakening the group constraint. 
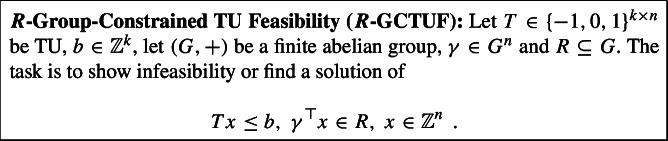
 Here, we typically call *R* the set of target elements. The above setup allows us to measure progress between GCTUF (the case of $|R|=1$) and an unconstrained IP with TU constraint matrix (captured by setting $R = G$). In particular, the difficulty of an $R$-GCTUF problem increases as the size of *R*, i.e., the number of target elements, decreases. The main parameter capturing this hardness is the *depth* $d:==|G|-|R|$ of the problem. We show the following generalization of Theorem 5.

### Theorem 12

Let *G* be a finite abelian group. There is a strongly polynomial randomized algorithm solving $R$-GCTUF problems with group *G* and $|G|- |R|\le 3$.

Our approach exploits Seymour’s decomposition theorem for TU matrices. To state this result, we first introduce the additional notions of a 3-sum of matrices, and pivoting operations (for more details on TU matrices and their decomposition, see, e.g., [35]).

### Definition 13

$(3-sum)$ Let $A\in \mathbb {Z}^{k_A\times n_A}$, $B\in \mathbb {Z}^{k_B\times n_B}$, $e\in \mathbb {Z}^{k_A}$, $f\in \mathbb {Z}^{n_B}$, $g\in \mathbb {Z}^{k_B}$, $h\in \mathbb {Z}^{n_A}$. The 3-*sum* of $$\left( {\begin{matrix} A &  e &  e \\ h^\top &  0 &  1 \end{matrix}}\right) $$ and $$\left( {\begin{matrix} 0 &  1 &  f^\top \\ g &  g &  B \end{matrix}}\right) $$ is $$\left( {\begin{matrix} A &  e &  e \\ h^\top &  0 &  1 \end{matrix}}\right) \oplus _{3} \left( {\begin{matrix} 0 &  1 &  f^\top \\ g &  g &  B \end{matrix}}\right) :==\left( {\begin{matrix} A &  ef^\top \\ gh^\top &  B \end{matrix}}\right) $$.

### Definition 14

*(Pivoting)* Let $C\in \mathbb {Z}^{k\times n}$, $p\in \mathbb {Z}^n$, $q\in \mathbb {Z}^k$, and $\varepsilon \in \{-1,1\}$. The matrix obtained from pivoting on $\varepsilon$ in $$T :==\left( {\begin{matrix} \varepsilon &  p^\top \\ q &  C \end{matrix}}\right) $$, i.e., pivoting on the element $T_{11}$ of *T*, is $${\text {pivot}}_{11}(T) :==\left( {\begin{matrix} -\varepsilon &  \varepsilon p^\top \\ \varepsilon q &  C - \varepsilon q p^\top \end{matrix}}\right) $$. More generally, ${\text {pivot}}_{ij}(T)$ for indices *i* and *j* such that $T_{ij}\in \{-1,1\}$ is obtained from *T* by first permuting rows and columns such that the element $T_{ij}$ is permuted to the first row and first column, then performing the above pivoting operation on the permuted matrix, and finally reversing the row and column permutations.

With this notation at hand, we can state Seymour’s TU decomposition theorem as follows.

### Theorem 15

(Seymour’s TU decomposition [14]) Let $T\in \mathbb {Z}^{k\times n}$ be a totally unimodular matrix. Then, one of the following cases holds.

(i) *T* or $T^\top$ is a network matrix.
(ii) *T* is, possibly after iteratively applying the operations of
  Deleting a row or column with at most one non-zero entry,
  Deleting a row or column that appears twice or whose negation also appears in the matrix, and
  Changing the sign of a row or column,
  equal to one of
  $$\begin{aligned} \left( {\begin{matrix} 1 &  -1 &  0 &  0 &  -1 \\ -1 &  1 &  -1 &  0 &  0 \\ 0 &  -1 &  1 &  -1 &  0 \\ 0 &  0 &  -1 &  1 &  -1 \\ -1 &  0 &  0 &  -1 &  1 \end{matrix}}\right) \quad \text {and}\quad \left( {\begin{matrix} 1 &  1 &  1 &  1 &  1 \\ 1 &  1 &  1 &  0 &  0 \\ 1 &  0 &  1 &  1 &  0 \\ 1 &  0 &  0 &  1 &  1 \\ 1 &  1 &  0 &  0 &  1 \end{matrix}}\right) \hspace{5.0pt}. \end{aligned}$$
(iii) *T* can, possibly after row and column permutations and pivoting once, be decomposed into a 3-sum of totally unimodular matrices with $n_A, n_B\ge 2$.

Additionally, we can in time $\textrm{poly}(n)$ decide which of the cases holds and determine the involved matrices.

The decomposition theorem in its original formulation imposes the less strict bound $n_A+n_B \ge 4$ in (iii). The above stated strengthening of the bound can be found in [1, 37].

We remark that typically, a 3-sum of the form $$\left( {\begin{matrix} A &  ef^\top \\ gh^\top &  B \end{matrix}}\right) $$ would be called a 1- or 2-*sum* if both or one of the off-diagonal blocks $ef^\top$ and $gh^\top$ were zero, respectively. However, as we treat those special cases in the same way as 3-sums, there is no need for us to further distinguish between them. Generally, we refer to matrices covered by Items (i) and (ii) of Theorem 15 as *base block* matrices. By porting results on congruency-constrained base block problems of [3] to the group-constrained setting and combining them with our new Theorem 7, it follows that GCTUF problems can be solved in strongly polynomial time if the constraint matrix is a base block matrix. For the sake of completeness, in Appendix A we comment on how to extend the arguments from [3] for base block problems arising from a network matrix or a special constant size matrix (i.e., Item (ii) and the first part of Item (i) from Theorem 15).

The potential pivoting step in Item (iii) of Theorem 15 can also be handled by extending a result from [3] to the group setting. For the sake of completeness, we sketch the extension in the following.

For congruency-constrained TU problems, [3, Theorem 2.7] shows that pivoting operations can be dealt with in the following sense: After the addition of a single variable upper bound to a given congruency-constrained TU problem, there is a unimodular variable transformation that transforms the problem into an equivalent congruency-constrained TU problem such that (up to one extra constraint that is an upper bound on a variable), the new constraint matrix is the desired pivoted form of the original constraint matrix.

The transformation argument sketched above only exploits that congruency constraints are constraints on a linear combination of the variables, and thus immediately extends to group constraints. Adding an upper bound constraint on a variable can be done without changing the problem due to a proximity result for congruency-constrained TU problems [3, Lemma 3.2]. The latter is based on a decomposition theorem for solutions of TU systems [3, Lemma 2.1] and the properties of cyclic groups that generalize to general finite abelian groups, and thus translates to group constraints.

Showing how to deal with $R$-GCTUF problems with constraint matrices that are 3-sums will lead to the following generalization of Theorem 6 which, in combination with the aforementioned results on base block problems, immediately implies Theorem 12. We devote the rest of this section to a discussion of its proof.

### Theorem 16

Let *G* be a finite abelian group and $\ell \in \mathbb {Z}_{\ge 1}$ with $\ell \ge |G|-3$. Given an oracle for solving base block $R$-GCTUF problems with group *G* and any $R\subseteq G$ with $|R|\ge \ell$, we can solve $R$-GCTUF problems with group *G* and $R\subseteq G$ with $|R| \ge \ell$ in strongly polynomial time with strongly polynomially many calls to the oracle.

### Reducing to a simpler problem when the target elements form a union of cosets

If *R*, the set of target elements, is a union of cosets of the same non-trivial proper subgroup *H* of *G* (i.e., it is of the form $R=\bigcup _{i=1}^k (g_i+H)$ for some $g_1,\ldots ,g_k\in G$, or equivalently, $R=R+H$), we can directly reduce to a simpler problem. We formalize this in the following lemma.

#### Lemma 17

Assume we are given an $R$-GCTUF problem

$$ Tx \le b,\ \gamma ^\top x \in R,\ x\in \mathbb {Z}^n $$

such that $R=R+H$ for a non-trivial proper subgroup *H* of *G*. Then, the set of feasible solutions of the given $R$-GCTUF problem is invariant under replacing *G* by the quotient group $\widehat{G} = G/H$, *R* by $\widehat{R} = R/H$, and $\gamma$ by its image $\widehat{\gamma }\in \widehat{G}^n$ under the quotient map.

#### Proof

Let *P* denote the original $R$-GCTUF problem, and let $\widehat{P}$ denote the modified one. The inequality system $Tx\le b$ is the same in *P* and $\widehat{P}$, hence it is enough to show that $\gamma ^\top x \in R$ if and only if $\widehat{\gamma }^\top x \in \widehat{R}$.

To this end, first note that for $x\in \mathbb {Z}^n$, $\gamma ^\top x \in R$ immediately implies $\widehat{\gamma }^\top x \in \widehat{R}$ by definition. For the other direction, assume $x\in \mathbb {Z}^n$ satisfies $\widehat{\gamma }^\top x \in R$. Then, by definition of $\widehat{\gamma }$ and $\widehat{R}$, we know that there is an element $h\in H$ such that $\gamma ^\top x + h \in R$. Then $\gamma ^\top x \in R - h = R$, as desired. $\square$

The depth of the new problem given by Lemma 17 in the corresponding hierarchy is $\widehat{d}=|G/H| - |R/H| = \frac{|G| - |R|}{|H|} < |G| - |R|$, so we indeed end up with a simpler problem in that respect. Since the existence of such a subgroup *H* can be checked efficiently (given that *G* has constant size), we can always and in constant time determine upfront whether the $R$-GCTUF problem at hand is reducible using Lemma 17, and if so, reduce it to a simpler $R$-GCTUF problem. Thus, for the rest of this section, we assume *R* is not a union of cosets. This assumption allows us to apply a special case of the Cauchy-Davenport theorem that holds despite the fact that the group order may not be prime. We refer to Lemma 20 for details.

### Decomposing the problem

We now focus on an $R$-GCTUF problem with a constraint matrix *T* that can be decomposed into a 3-sum of the form $$T=\left( {\begin{matrix}A &  ef^\top \\ gh^\top &  B\end{matrix}}\right) $$. The decomposition allows for splitting *x*, *b*, and $\gamma$ into two parts accordingly, giving the equivalent formulation

2 $$\begin{aligned} \begin{pmatrix} A &  ef^\top \\ gh^\top &  B \end{pmatrix} \cdot \begin{pmatrix} x_A \\ x_B \end{pmatrix} \le \begin{pmatrix} b_A \\ b_B \end{pmatrix}\hspace{5.0pt},\quad \gamma _A^\top x_A + \gamma _B^\top x_B \in R\hspace{5.0pt},\quad \begin{aligned} x_A&\in \mathbb {Z}^{n_A} \\ x_B&\in \mathbb {Z}^{n_B} \end{aligned}\hspace{5.0pt}. \end{aligned}$$

In the inequality system, the variables $x_A$ and $x_B$ interact only through the rank-one blocks $ef^\top$ and $gh^\top$. Fixing values of $\alpha :==f^\top x_B$ and $\beta :==h^\top x_A$ allows for rephrasing (2) through the following two almost independent problems

3 $$\begin{aligned} \begin{array}{lll} A x_A \le b_A - \alpha e & \quad &  \quad \quad B x_B \le b_B - \beta g\\ h^\top x_A = \beta & \quad \text {and}& \quad f^\top x_B = \alpha \\ x_A \in \mathbb {Z}^{n_A} & \quad &  \quad \quad \quad x_B \in \mathbb {Z}^{n_B}\quad \quad , \end{array} \end{aligned}$$

where we seek to find solutions $x_A$ and $x_B$ such that their corresponding group elements $r_A:==\gamma _A^{\top } x_A$ and $r_B:==\gamma _B^{\top } x_B$, respectively, satisfy $r_A + r_B \in R$. Hence, this desired relation between the target elements $r_A$ and $r_B$ is the only dependence between the two problems once $\alpha$ and $\beta$ are fixed. We assume without loss of generality that *A* has no fewer columns than *B*, and refer to the problem on the left as the *A-problem*, and the problem on the right as the *B-problem*. We denote by $\Pi$ the set of all $(\alpha , \beta )\in \mathbb {Z}^{2}$ such that both the *A*- and *B*-problem are feasible. (Note that both problems are described through a TU constraint matrix; hence, feasibility can be checked efficiently.) Also, for $(\alpha ,\beta )\in \Pi$, let $\pi _A(\alpha ,\beta ) \subseteq G$ be all group elements $r_A\in G$ for which there is a solution $x_A$ to the *A*-problem with $\gamma ^\top x_A = r_A$, and define $\pi _B$ analogously. We refer to $\pi _A$ and $\pi _B$ as *patterns*. Hence, (2) is feasible if and only if there is a pair $(\alpha ,\beta )\in \Pi$ such that, for some $r_A\in \pi _A(\alpha ,\beta )$ and $r_B \in \pi _B(\alpha ,\beta )$, we have $r_A+r_B\in R$. Thus, patterns contain all information needed to decide feasibility.

Using techniques from [3], we can restrict our search for feasible solutions to a constant-size subset $\widehat{\Pi } \subseteq \Pi$. More precisely, one can show the following.

#### Lemma 18

One can in strongly polynomial time find $\ell _i, u_i\in \mathbb {Z}$ for $i\in \{0,1,2\}$, with $u_i-\ell _i\le d$ such that

4 $$\begin{aligned} \widehat{\Pi } :==\left\{ (\alpha , \beta )\in \mathbb {Z}^2 :\ell _0 \le \alpha +\beta \le u_0, \ell _1 \le \alpha \le u_1, \ell _2\le \beta \le u_2\right\} \end{aligned}$$

satisfies $\widehat{\Pi }\subseteq \Pi$, and if (2) is feasible, then there is a pair $(\alpha ,\beta )$ in $\widehat{\Pi }$ for which there is a solution $x_A$ to the *A*-problem and a solution $x_B$ to the *B*-problem with $\gamma ^\top x_A + \gamma ^\top x_B \in R$.

This statement was proved in the congruency constraint setting in [3]. To obtain the result, [3] exploits a decomposition theorem for solutions of totally unimodular systems [3, Lemma 2.1] combined with the following fact, which was only stated for the special case of $G=\mathbb {Z}/m\mathbb {Z}$ [3, Lemma 2.2], and which is indeed the only property of $\mathbb {Z}/m\mathbb {Z}$ used throughout the proof.

#### Lemma 19

Let *G* be a finite abelian group, $R\subseteq G$, and $r_1,\ldots ,r_\ell \in G$ with $\sum _{i\in [\ell ]} r_i \in R$. If there is no interval $I=\{i_1,\ldots ,i_2\}$ with $i_1,i_2\in [\ell ]$ and $i_1<i_2$ such that $\sum _{i\in [\ell ]{\setminus } I}r_i\in R$, then $\ell \le |G|-|R|$.

Consequently, through Lemma 19, the proofs of [3, Lemmas 2.5 and 5.1] immediately extend to the group constraint setting, and thereby imply Lemma 18. We also remark that Lemma 19 is in fact just a slightly generalized and more constructive version of Lemma 11. Moreover, the original proof for $G=\mathbb {Z}/m\mathbb {Z}$ directly generalizes. We repeat it here for completeness.

#### Proof of Lemma 19

Assume for the sake of deriving a contradiction that there is no interval $I\subseteq [\ell ]$ with $\sum _{i\in [\ell ]{\setminus } I}r_i\in R$, but $\ell \ge |G|-|R|+1$. Consider the $\ell$ group elements $s_0=0$, $s_1=r_1$, ..., $s_{\ell -1}=r_1+\ldots +r_{\ell -1}$. Observe that $s_j\notin R$ for all $j\in [\ell -1]$; for otherwise, there is an interval $I=\{j+1,\ldots ,\ell \}$ for some $j\in [\ell -1]$ such that $\sum _{i\in [\ell ]\setminus I} = s_j\in R$, contradicting the assumption. Thus, $s_j\in G{\setminus } R$ for all $j\in [\ell -1]$. Hence, because $\ell \ge |G|-|R|+1$, we have by the pigeonhole principle that there exist $j_1,j_2\in [\ell -1]$ with $j_1<j_2$ such that $s_{j_1}=s_{j_2}$. Thus, $I=\{j_1+1, \ldots , j_2\}$ is an interval with $\sum _{i\in [\ell ]{\setminus } I} = \sum _{i\in [\ell ] I}r_i - (s_{j_2}-s_{j_1}) = \sum _{i\in [\ell ]{\setminus } I} r_i \in R$, again contradicting the assumption and hence completing the proof. $\square$

Considering Lemma 18, the challenges lie less in the size of $\Pi$, but rather in how to obtain information on the sets $\pi _A(\alpha ,\beta )$ and $\pi _B(\alpha ,\beta )$ for pairs $(\alpha ,\beta )\in \Pi$. Opposed to previous techniques, which almost solely focused on $\pi _B$, we investigate both $\pi _A$ and $\pi _B$ and their interplay—see Sect. 4.3.

As *B* has at most half the columns of the constraint matrix *T* of the original $R$-GCTUF problem (2), we can afford (runtime-wise) to recursively call our algorithm multiple times on the *B*-problem for different targets $R_B$ of the same depth $d=|G|-|R|$ as the original problem, i.e., with $|R_B|=|R|$. (We refrain from using larger depths, as GCTUF become harder with increasing depth.) This allows us to compute a set $\bar{\pi }_B(\alpha ,\beta )\subseteq \pi _B(\alpha ,\beta )$ of size $|\bar{\pi }_B(\alpha ,\beta ))|=\min \{d+1,\pi _B(\alpha ,\beta )\}$. Indeed, we can start with $\bar{\pi }_B(\alpha ,\beta )=\emptyset$ and, as long as $|\bar{\pi }_B(\alpha ,\beta )| < \min \{d+1,\pi _B(\alpha ,\beta )\}$, we solve an $R_B$-GCTUF *B*-problem (i.e., we look for a *B*-problem solution $x_B$ with $\gamma ^\top x_B \in R_B$) with $R_B = G{\setminus } \bar{\pi }_B(\alpha ,\beta )$ being a set of size at least $|G|-d$. If $R_B\cap \pi _B(\alpha ,\beta )\ne \emptyset$, then we find an element in $R_B\cap \pi _B(\alpha ,\beta )$ that can be added to $\bar{\pi }_B(\alpha ,\beta )$ and we repeat; otherwise, $R_B\cap \pi _B(\alpha ,\beta )=\emptyset$ and we know that we computed $\bar{\pi }_B(\alpha ,\beta )=\pi _B(\alpha ,\beta )$.

To the contrary, note that the *A*-problem may be almost as big as the original GCTUF problem (possibly with just two fewer columns). Hence, here we cannot afford (runtime-wise) a similar computation as for the *B*-problem. However, we can afford to solve multiple $R_A$-GCTUF *A*-problems of smaller depth, i.e., $|R_A|>|R|$, because the runtime decreases significantly with decreasing depth. By using the same approach as in the *B*-problem, but with sets $R_A$ of size $|R_A|\ge |R|+1$, we obtain a set $\bar{\pi }_A(\alpha ,\beta )\subseteq \pi _A(\alpha ,\beta )$ of size $|\bar{\pi }_A(\alpha ,\beta )|=\min \{d,\pi _A(\alpha ,\beta )\}$.

Let us next take a closer look at patterns. Fix some $(\alpha ,\beta )\in \Pi$ and let $\pi _A (\alpha ,\beta )= \{r_A^1,\ldots ,r_A^{\ell _A}\}$ for some $\ell _A \ge 1$ and pairwise different $r_A^i\in G$, and let $x_A^{1},\ldots , x_A^{\ell _A}$ be corresponding solutions of the *A*-problem with $\gamma _A^\top x_A^i=r_A^i$. Define $\ell _B$, $r_B^i$, and $x_B^i$ analogously. Observe that if $\ell _A\le d$ and $\ell _B\le d+1$, we have $\bar{\pi }_X(\alpha ,\beta )= \pi _X(\alpha ,\beta )$ for both $X\in \{A,B\}$. Hence, we can compute all feasible group elements and check explicitly whether $r_A^i+r_B^j\in R$ for some $i\in [\ell _A]$ and $j\in [\ell _B]$, i.e., whether a solution exists. If $\ell _B\ge d+1$, we can (independently of $\ell _A$) even show that there always exists a feasible solution, and we can also find one: Indeed, we can compute $d+1$ solutions $x^i:==(x^1_A, x^i_B)$ with pairwise different sums $r^1_A + r^i_B\in G$, at least one of which must satisfy $r^1_A + r^i_B \in R$. If $\ell _A\ge d$ and $\ell _B\ge 2$, we can argue similarly: We show that among any *d* elements of $\bar{\pi }_A (\alpha ,\beta )$, and any two elements of $\bar{\pi }_B (\alpha ,\beta )$ (which we can compute), there is a pair $r^i_A, r^j_B$ with $r^i_A + r^j_B \in R$. Note that while for groups of prime order this can be shown via the Cauchy-Davenport theorem, the above result does not hold in general. We show, however, that as long as *R* is not a union of cosets in *G*, we can recover the implication (cf. Section 4.1 for why this assumption is legit).

#### Lemma 20

Let *G* be a finite abelian group, and let $R \subseteq G$ be such that $R \ne R+H$ for any non-trivial subgroup *H* of *G*. Then, for any subsets $X,Y\subseteq G$ with $|X| = |G|-|R|$ and $|Y|\ge 2$, we have $(X+Y)\cap R \ne \emptyset$.

#### Proof

Let $b_1, b_2 \in Y$ with $b_1\ne b_2$, and set $h= b_1-b_2$. Assume $\left( X + Y\right) \cap R = \emptyset$. Then $|X| = |G| - |R|$ implies $|X + Y| =|X|$. Thus, $X + b_1 = X + b_2$ and hence $X = X + h$. Iterating gives $X = X + \langle h \rangle$, where $\langle h \rangle$ denotes the subgroup generated by *h*. As $R = G {\setminus } (X + b_1)$, we get $R = R + \langle h \rangle$, a contradiction. $\square$

The following observation summarizes the above discussion.

#### Observation 21

Let $(\alpha ,\beta )\in \widehat{\Pi }$. If $|\bar{\pi }_A (\alpha ,\beta )|\le d-1$ or $|\bar{\pi }_B (\alpha ,\beta )|\ge 2$, we can immediately determine whether a feasible solution to the original $R$-GCTUF problem exists for such $(\alpha ,\beta )$, and if so, obtain one by combining solutions computed for the *A*- and *B*-subproblem when determining $\bar{\pi }_A$ and $\bar{\pi }_B$.

Thus, the only case in which we cannot immediately check whether a feasible solution exists for some $(\alpha ,\beta )$, is when $\ell _B=1$ and $\ell _A \ge d+1$ (which imply $|\bar{\pi }_A (\alpha ,\beta )| = d$ and $|\bar{\pi }_B (\alpha ,\beta )|= 1$). This is the only case where we may have $\left( \pi _A (\alpha ,\beta )+ \pi _B (\alpha ,\beta )\right) \cap R \ne \emptyset$ but $\left( \bar{\pi }_A (\alpha ,\beta )+ \bar{\pi }_B (\alpha ,\beta )\right) \cap R = \emptyset$, in which case we say that $(\alpha ,\beta )$ contains a *hidden solution*.

### New insights towards overcoming previous barriers for $d=3$

We now describe how our new techniques allow for overcoming barriers restricting previous approaches to depth $d=2$. Recall that we focus on a constant size subset $\widehat{\Pi }$ as defined in (4). We call sets of this form, for any choice of $\ell _i$ and $u_i$, *pattern shapes*, and denote by

5 $$\begin{aligned} \mathcal {D}:==\left\{ \pm \left( {\begin{matrix} 1 \\ 0 \end{matrix}}\right) , \pm \left( {\begin{matrix} 0 \\ 1 \end{matrix}}\right) , \pm \left( {\begin{matrix} 1 \\ -1 \end{matrix}}\right) \right\} \end{aligned}$$

the possible edge directions of ${\text {conv}}(\widehat{\Pi })$. Focusing on $\widehat{\Pi }$ allows for efficiently computing $\bar{\pi }_X(\alpha ,\beta )$ for $X\in \{A,B\}$ and all $(\alpha ,\beta )\in \widehat{\Pi }$ to the extent discussed earlier. In order to proceed, we use a structural result from [3], called averaging, that allows us to relate solutions—and thus elements of $\pi _X$—across different $(\alpha ,\beta )$. Despite being true in more generality, the exposition here requires the following special case only.

#### Proposition 22

( [3, special case of Lemma 5.3]) Consider an $R$-GCTUF problem as described in (2). Let $X \in \{A,B\}$, $v\in \mathcal {D}$, and $(\alpha ,\beta )\in \widehat{\Pi }$ with $(\alpha ,\beta )+ 2v \in \widehat{\Pi }$. Given a solution $x_1$ of the *X*-problem for $(\alpha ,\beta )$ and, similarly, $x_2$ for $(\alpha ,\beta )+2v$, there are solutions $x_3,x_4$ for the *X*-problem for $(\alpha ,\beta )+ v$ such that $x_1+x_2 = x_3+x_4$.

We remark that the proof of the above result for congruency-constrained problems given in [3] only exploits that congruency-constraints are linear constraints; therefore, the result carries over to group-constraints seamlessly.

**Fig. 1:**
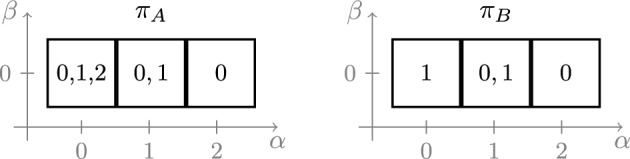
Possible patterns $\pi _A$ and $\pi _B$ for a problem with group $G=\mathbb {Z}/4\mathbb {Z}$. Every square corresponds to a pair $(\alpha ,\beta )\in \widehat{\Pi }$, and the numbers in the box indicate elements of $\pi _A(\alpha ,\beta )$ and $\pi _B(\alpha ,\beta )$, respectively. For $R=\{3\}$, there is a feasible solution with $(\alpha ,\beta )=(0,0)$, but this cannot be detected without studying $\pi _A$

In previous approaches for depth $d=2$, it was enough to only compute a single element from $\pi _A$ (e.g., by solving the *A*-problem after dropping the group constraint). Concretely, consider patterns of the shape as given in Fig. 1. For $d=2$, Proposition 22 can be used to show that, if there is a hidden feasible solution for $(\alpha ,\beta )=(0,0)$ or $(\alpha ,\beta )=(2,0)$, then there must also be a feasible solution for $(\alpha ,\beta )=(1,0)$. The example in Fig. 1 shows that this is no longer true if the depth *d* exceeds 2, as only $(\alpha ,\beta )=(0,0)$ admits a feasible solution.

This problem can be circumvented by analyzing the *A*-pattern $\bar{\pi }_A$. As argued in Sect. 4.2, if a pair $(\alpha ,\beta )$ has a hidden solution, then $|\pi _A(\alpha ,\beta )| \ge d+1$ (and hence $|\bar{\pi }_A(\alpha ,\beta )| = d$), hence we assume that there exists at least one such pair. The following result uses averaging (i.e., Proposition 22) to show that pairs $(\alpha ',\beta ')$ adjacent to such a pair $(\alpha ,\beta )$ containing a hidden solution also have large $\bar{\pi }_A (\alpha ',\beta ')$.

#### Lemma 23

Let $d\in \{1,2,3\}$, $v \in \mathcal {D}$, and $(\alpha , \beta )\in \widehat{\Pi }$ such that $|\pi _A(\alpha ,\beta )|\ge d+1$ and $(\alpha , \beta )+2v\in \widehat{\Pi }$. Then $|\bar{\pi }_A((\alpha ,\beta )+v)| = d$.

#### Proof

It is enough to show that $|\pi _A((\alpha ,\beta )+v)|\ge d$. To this end, for each of the at least $d+1$ elements $r\in \pi _A(\alpha ,\beta )$, let $x_1^r$ be a corresponding solution of the *A*-problem, and let $x_2$ denote any fixed solution for the *A*-problem on the pair $(\alpha ,\beta )+2v$. Proposition 22 applied to $x_1^r$ and $x_2$ gives solutions $x_3^r$ and $x_4^r$ corresponding to elements $\gamma _A^\top x_3^r,\gamma _A^\top x_4^r\in \pi _A((\alpha ,\beta )+v)$ with $\gamma _A^\top x_3^r + \gamma _A^\top x_4^r$ taking at least $d+1$ different values. Assume for the sake of deriving a contradiction that $|\pi _A((\alpha ,\beta )+v)|\le d-1$. Then, since the number of different sums of pairs of elements in $\pi _A((\alpha ,\beta )+v)$ is bounded by $$\left( {\begin{array}{c}d-1\\ 2\end{array}}\right) +d - 1 = (d-1)d/2< d+1 $$ for $d\in \{1,2,3\}$, this contradicts the above construction. $\square$

#### Remark 24

**Fig. 2:**
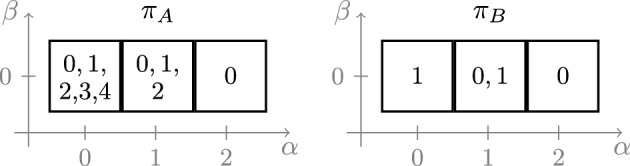
Possible patterns $\pi _A$ and $\pi _B$ for a problem with group $G=\mathbb {Z}/5\mathbb {Z}$. Every square corresponds to a pair $(\alpha ,\beta )\in \widehat{\Pi }$, and the numbers in the box indicate the elements of $\pi _A(\alpha ,\beta )$ and $\pi _B(\alpha ,\beta )$, respectively. For $d=4$, Lemma 23 fails to hold for $(\alpha ,\beta )=(0,0)$ and $v=(1,0)$

For depth $d=4$, one can find GCTUF problems with $G=\mathbb {Z}/5\mathbb {Z}$ and patterns that fail to satisfy Lemma 23; we present one such example in Fig. 2. Thus, the approach used in this paper does not directly extend to $\Delta > 4$. We remark that Lemma 23 is the only place in our proofs where we use the assumption that $d = |G|-|R| \le 3$.

To proceed, we observe that if, on top of the assumption in Lemma 23, $|\pi _B((\alpha ,\beta )+v)| \ge 2$ holds, then Lemma 20 guarantees $(\bar{\pi }_A((\alpha ,\beta )+v) + \bar{\pi }_B((\alpha ,\beta )+v)) \cap R \ne \emptyset$, i.e., existence of a feasible solution. Thus, from now on, we analyze both the *A*- and *B*-patterns in detail, in particular through averaging, to find a pattern constellation as mentioned above, or identify additional properties that allow for direct progress.

### Analyzing pattern structure

**Fig. 3:**
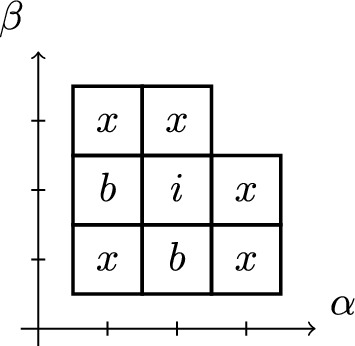
A pattern shape with interior, border, and vertex pairs (marked *i*, *b*, and *x*, respectively)

Before getting to an exhaustive analysis of patterns based on the insights laid out earlier, we introduce notions that will allow us to distinguish patterns from a structural point of view (also see Fig. 3).

#### Definition 25

Let $\mathcal {D}$ be the possible edge directions of a pattern shape as defined in (5). We call $(\alpha , \beta )\in \widehat{\Pi }$ an *interior pair* if $(\alpha , \beta ) + v \in \widehat{\Pi }$ for all $v\in \mathcal {D}$, a *border pair* if $(\alpha , \beta ) \pm v \in \widehat{\Pi }$ for exactly two $v\in \mathcal {D}$, and a *vertex pair* if it is not an interior or border pair.

Note that for a border pair $(\alpha ,\beta )$, due to symmetry, the two directions $v\in \mathcal {D}$ satisfying $(\alpha ,\beta )\pm v\in \widehat{\Pi }$ will always be antiparallel, i.e., *v* and $-v$ for some $v\in \mathcal {D}$.

Next, we summarize results of [3] that we reuse here. We remark that these results were proved in a congruency-constrained setting, but translate to problems with group constraints straightforwardly. For the sake of completeness, we recall proof ideas and comment on how to adapt them to the group constraint setting that we work with.

#### Theorem 26

( [3]) Consider an $R$-GCTUF problem as described in (2).

(i) If there is some $(\alpha ,\beta )\in \widehat{\Pi }$ with $|\bar{\pi }_B(\alpha ,\beta )| \ge 2$, then for each $(\alpha ',\beta ')\in \widehat{\Pi }$, there exists $v\in \mathcal {D}\cup \{0\}$ such that $|\bar{\pi }_B((\alpha ',\beta ')+v)| \ge 2$. If in addition, $\widehat{\Pi }$ contains an interior pair, then for each $(\alpha ',\beta ')\in \widehat{\Pi }$, *v* can be chosen such that we additionally have $(\alpha ',\beta ')+2v \in \widehat{\Pi }$.
(ii) If $|\pi _B(\alpha ,\beta )|= 1$ for all $(\alpha ,\beta )\in \widehat{\Pi }$, or $\widehat{\Pi }$ only contains vertex pairs and there are no solutions for $(\alpha ,\beta )\in \widehat{\Pi }$ with $|\bar{\pi }_B(\alpha ,\beta )|\ge 2$, then the problem can be reduced to a single $R$-GCTUF problem with the same group *G* and at the same depth *d*, and strictly fewer variables.

#### Proof

The statements in Theorem 26 are closely linked to a GCTUF problem in the form presented in (2). In the following, we say that a solution $x=(x_A, x_B)$ of a problem in that form is *a solution for* $(\alpha ,\beta )$ if $\alpha = f^\top x_B$ and $\beta = h^\top x_A$.

In the congruency-constrained setting, the first part of Theorem 26 (i) was proved in [3, Lemma 5.9], while the second part is implicit in [3, Proof of Lemma 5.8]. The argument uses a generalized version of Proposition 22, which states that given distinct pairs $(\alpha _{1}, \beta _{1}), (\alpha _{2}, \beta _{2})\in \widehat{\Pi }$, there are $(\alpha _{3}, \beta _{3}), (\alpha _{4}, \beta _{4})\in \widehat{\Pi }$ such that for any solutions $x_1$ for $(\alpha _{1}, \beta _{1})$ and $x_2$ for $(\alpha _{2}, \beta _{2})$, there exist solutions $x_3$ for $(\alpha _{3}, \beta _{3})$ and $x_4$ for $(\alpha _{4}, \beta _{4})$ such that $x_1+x_2= x_3+x_4$ (in fact, $(\alpha _{3}, \beta _{3})$ and $(\alpha _{4}, \beta _{4})$ are equal to $\frac{1}{2}((\alpha _{1}, \beta _{1})+(\alpha _{2}, \beta _{2}))$ up to rounding, see [3, Lemma 5.3]).

Towards a proof of Theorem 26 (i), we may assume that $(\alpha ',\beta ')\ne (\alpha ,\beta )+ v$ for all $v\in \mathcal {D}\cup \{0\}$, and apply the above with $(\alpha _{1}, \beta _{1})=(\alpha ',\beta ')$ and $(\alpha _{2}, \beta _{2})=(\alpha ,\beta )$. This gives that $(\alpha _{3}, \beta _{3})$ and $(\alpha _{4}, \beta _{4})$ are both different from $(\alpha _{1}, \beta _{1})$ and $(\alpha _{2}, \beta _{2})$, but “closer” to $(\alpha ',\beta ')$ than $(\alpha ,\beta )$ was. The result will follow by iteratively applying this argument after showing that $|\pi _B(\alpha _{3}, \beta _{3})|\ge 2$ or $|\pi _B(\alpha _{4}, \beta _{4})|\ge 2$. In the congruency-constrained case, the latter is concluded from the assumption that $|\pi _B (\alpha ,\beta )|\ge 2$, i.e., that there are two feasible residues at $(\alpha ,\beta )$. Indeed, if we had $|\pi _B(\alpha _{3}, \beta _{3})|=|\pi _B(\alpha _{4}, \beta _{4})|=1$, then $\gamma ^\top (x_3+x_4)$—and hence also $\gamma ^\top (x_1+x_2)$—would have to yield the same residue for all solutions $x_1$ for $(\alpha _{1}, \beta _{1})$ and $x_2$ for $(\alpha _{2}, \beta _{2})$. This reasoning holds analogously for any finite abelian group other than the cyclic groups $\mathbb {Z}/m\mathbb {Z}$, by well-definedness of addition to be precise. Because no other properties of congruency-constraints are exploited, the proofs of [3] directly translate to a proof of Theorem 26 (i).

Theorem 26 (ii) gives two sufficient conditions that allow for reduction to a problem with fewer variables. The conditions are precisely that the pattern structure is of (I), or that it is of (IV) and that there are no solutions for pairs $(\alpha ,\beta )\in \widehat{\Pi }$ with $|\pi (\alpha ,\beta )|\ge 2$.

For pattern structure of (I), [3, Corollary 5.1] shows that—in the congruency-constrained setting—there exist $r_0, r_1, r_2\in \mathbb {Z}/m\mathbb {Z}$ such that $\pi _B(\alpha ,\beta )= \{r_0 + r_1\alpha + r_2\beta \}$ for all $(\alpha ,\beta )\in \widehat{\Pi }$, and $\pi _B$ is called *linear* in this case. Again, the proof is based on an averaging argument (i.e., Proposition 22) that seamlessly carries over to the more general group-constrained setting with a finite abelian group *G* by simply replacing calculations in $\mathbb {Z}/m\mathbb {Z}$ by calculations in *G*. The same applies to showing that in case of a linear pattern, a GCTUF problem can be reduced to an equivalent problem with the same group *G*, at the same depth *d*, and strictly fewer variables [3, Theorems 2.4 and 2.5].

For pattern structure of (IV), Lemma 27 gives that $\widehat{\Pi }$ consists of at most four pairs, and all of them are vertex pairs. This pattern family is very restricted, and is in fact a subset of the patterns covered by an analysis of certain small patterns in [3, Proof of Lemma 5.11]. Concretely, for the (IV) pattern structure that we consider here, it was concluded (using another averaging argument, i.e., Proposition 22) that one can choose for every $(\alpha ,\beta )\in \widehat{\Pi }$ a singleton-subset $\tilde{\pi }_B(\alpha ,\beta )\subseteq \pi _B(\alpha ,\beta )$ such that $\tilde{\pi }_B$ is linear in the sense introduced above. Now recall that we also assume here that there are no solutions for pairs $(\alpha ,\beta )\in \widehat{\Pi }$ with $|\pi (\alpha ,\beta )|\ge 2$, i.e., solutions can only occur for $(\alpha ,\beta )\in \widehat{\Pi }$ with $|\pi _B(\alpha ,\beta )|=1$. For those $(\alpha ,\beta )$, we have $\pi _B(\alpha ,\beta )=\tilde{\pi }_B(\alpha ,\beta )$, so it is enough to look for solutions compatible with $\tilde{\pi }_B$. But then, linearity of $\tilde{\pi }_B$ allows for the same reduction to an equivalent problem with fewer variables as discussed above (the congruency-constrained version is given in [3, Theorems 5.2 and 2.5]). Again, in all involved proofs of [3], all calculations in $\mathbb {Z}/m\mathbb {Z}$ can directly be replaced by calculations in any fixed finite abelian group *G* without affecting correctness of the proofs, hence the results carry over as desired. $\square$

The two statements in the above theorem serve a complimentary purpose: While Item (ii) allows for direct progress (by reducing the number of variables), particularly in the case where no $(\alpha ,\beta )\in \widehat{\Pi }$ satisfies $|\pi _B(\alpha ,\beta )|\ge 2$, Item (i) shows that whenever such $(\alpha ,\beta )$ are present, then they are, in a certain sense, well spread over the pattern. We will exploit the latter in combination with Lemma 23.

To formally analyze patterns, based on Observation 21, we may assume that we face an $R$-GCTUF problem for which $\widehat{\Pi }$ contains at least one $(\alpha ,\beta )$ such that $|\pi _A (\alpha ,\beta )|\ge d+1$ and $|\pi _B (\alpha ,\beta )|=1$. Starting from there, we distinguish four different types of pattern structure as follows:

(I) $|\pi _B(\alpha ,\beta )|=1$ for all $(\alpha ,\beta )\in \widehat{\Pi }$, or this is not the case and
(II) $\widehat{\Pi }$ has an interior pair, or
(III) $\widehat{\Pi }$ has no interior but border pairs, or
(IV) $\widehat{\Pi }$ has only vertex pairs.

Note that the case distinction above depends on $\pi _B$, which we do not fully know. Nonetheless, we can distinguish among the four types, as we can determine $\bar{\pi }_B$ and we have that $\pi _B(\alpha ,\beta )=\bar{\pi }_B(\alpha ,\beta )$ whenever $|\pi _B(\alpha ,\beta )|\le d+1$. Thus, we can in particular distinguish the case $|\pi _B(\alpha ,\beta )|=1$ and $|\pi _B(\alpha ,\beta )|\ge 2$ when observing $\bar{\pi }(\alpha ,\beta )$, allowing us to identify patterns of (I). Finally, to distinguish patterns of (II), (III), and (IV), note that we explicitly know $\widehat{\Pi }$, and hence we can check which kinds of pairs it contains.

The remainder of this section is devoted to presenting how to achieve progress in each of these four cases.

#### Pattern structure of (I): $|\pi _B(\alpha ,\beta )|=1$ for all $(\alpha ,\beta )\in \widehat{\Pi }$

Pattern structure of (I) is covered by Theorem 26 (ii), which allows to reduce the problem to a new GCTUF problem with same group *G* and same depth *d*, and at least one variable less, thus allowing to make progress in that respect.

#### Pattern structure of (II): $\widehat{\Pi }$ has an interior pair

For pattern structure of (II), we argue that if the $R$-GCTUF problem is feasible, then $\left( \bar{\pi }_A + \bar{\pi }_B\right) \cap R \ne \emptyset$. More precisely, we show that there must exist $(\alpha ,\beta )\in \widehat{\Pi }$ with $|\bar{\pi }_A (\alpha ,\beta )| = d$ and $|\bar{\pi }_B (\alpha ,\beta )| \ge 2$. This then implies the desired result by Lemma 20. Concretely, assume that there exist $(\alpha ', \beta ')\in \widehat{\Pi }$ containing a hidden solution. Then, since $\widehat{\Pi }$ contains an interior pair, and there exists $(\alpha ,\beta )\in \widehat{\Pi }$ with $|\pi _B (\alpha ,\beta )| \ge 2$, by Theorem 26 (i) there exists $v\in \mathcal {D}$ such that $(\alpha ', \beta ') + 2v \in \widehat{\Pi }$ and $|\pi _B((\alpha ', \beta ') + v)|\ge 2$. As Lemma 23 implies that $|\bar{\pi }_A((\alpha ', \beta ') + v)| = d$, it follows by Lemma 20 that $\left( \bar{\pi }_A ((\alpha ', \beta ')+v) + \bar{\pi }_B ((\alpha ', \beta ')+v) \right) \cap R \ne \emptyset$; thus we can find a solution at $(\alpha ',\beta ')+v$.

#### Pattern structure of (III): $\widehat{\Pi }$ has no interior but border pairs

In this case, we show that if $\bar{\pi }_A + \bar{\pi }_B$ does not hit the target set *R*, i.e., we fail to find a solution by combining solutions of the *A*- and *B*-problem that we computed recursively, then we can reduce to a smaller pattern shape $\Pi '$, and recurse. Through such a reduction, we will after constantly many steps reach pattern structures of (I) or (IV), and therefore achieve progress through the techniques presented for the corresponding type.

To start with, we show the following structural auxiliary result. We remark that this result can be seen as an implication of [3, Lemma 5.12], but we provide a more direct proof here.

##### Lemma 27

Assume that $\widehat{\Pi }$ does not contain interior pairs. Then $\widehat{\Pi }$ contains at most four vertex pairs.

##### Proof

Through shifting, we may assume that $\ell _1 = \ell _2 = 0$, and thus also $\ell _3\ge 0$ (if $\ell _3<0$, we may set it to zero without changing $\widehat{\Pi }$). Similarly, we may assume $u_3\le u_1+u_2$. Note that if $u_1 - \ell _1 \le 1$ there are at most four vertex pairs: At most two pairs $(\alpha ,\beta )$ may satisfy $\alpha = \ell _1$, and at most two further pairs may have $\alpha = u_1$. Similarly, we are done if $u_2 - \ell _2 \le 1$, or $u_3 - \ell _3\le 1$. Thus, we assume $u_i - \ell _i\ge 2$ for $i\in \{1,2,3\}$. Consider the pair

$$ (\alpha ,\beta )= {\left\{ \begin{array}{ll} (1, \ell _3) &  \text { if } \ell _3 < u_2\\ (\ell _3-u_2+2, u_2-1) &  \text { if } \ell _3 \ge u_2 \end{array}\right. }\hspace{5.0pt}. $$

By definition, $0<\alpha$, $\beta <u_2$, and $\ell _3< \ell _3+1 = \alpha +\beta < u_3$. Because $(\alpha ,\beta )$ can not be an interior pair, we must either have $\alpha \ge u_1$, or $\beta \le 0$. Because $u_1\ge 2+\ell _1=2$, we can only have $\alpha \ge u_1$ in the case $\ell _3\ge u_2$, which implies $\ell _3 + 2 \ge u_1+u_2$. As also, $\ell _3 + 2 \le u_3 \le u_1+u_2$, these inequalities must be tight, implying that there are precisely the three vertex pairs $(u_1, u_2), (u_1-2,u_2)$, and $(u_1, u_2-2)$. Similarly, because $u_2-1\ge 1+\ell _2\ge 1$, we can only have $\beta \le 0$ in the case $\ell _3<u_2$, which implies $\ell _3=0$. Consequently, we must also have $u_2=2$; otherwise (1, 1) is an interior pair. This implies that there are precisely the three vertex pairs (0, 0), (2, 0), (0, 2), and completes the proof. $\square$

With the above at hand, we can achieve the desired progress for pattern structure of (III). This is summarized in the following technical lemma.

##### Lemma 28

Consider an $R$-GCTUF instance of the form given in (2), and let the corresponding pattern shape $\widehat{\Pi }$ be of (III). Then either $\left( \bar{\pi }_A(\alpha ,\beta )+ \bar{\pi }_B(\alpha ,\beta )\right) \cap R \ne \emptyset$ for some $(\alpha ,\beta )\in \widehat{\Pi }$, or we can in strongly polynomial time find a pattern shape $\Pi ' \subsetneq \widehat{\Pi }$ such that the $R$-GCTUF instance is feasible on $\widehat{\Pi }$ if and only if it is feasible on $\Pi '$.

##### Proof

We distinguish two different cases based on the structure of border pairs in $\pi _B$.

- Case 1: There exists a border pair $(\alpha ,\beta )$ with $|\bar{\pi }_B(\alpha ,\beta )| \ge 2$. Consider the unique constraint in the inequality description of $\widehat{\Pi }$ that is tight at $(\alpha ,\beta )$. We claim that if there is a hidden solution at another pair $(\alpha ',\beta ')$ that satisfies the same constraint with equality, then we also see a solution in the computed patterns, i.e., $\left( \bar{\pi }_A(\bar{\alpha },\bar{\beta }) + \bar{\pi }_B(\bar{\alpha },\bar{\beta })\right) \cap R \ne \emptyset$ for some $(\bar{\alpha },\bar{\beta })\in \widehat{\Pi }$. Thus, if we do not find a solution right away by combining elements from $\bar{\pi }_A(\alpha ,\beta )$ and $\bar{\pi }_B(\alpha ,\beta )$, then there cannot be a hidden solution anywhere on the tight constraint under consideration. Thus, strengthening the tight constraint by one unit leads to the desired pattern shape $\Pi '\subsetneq \widehat{\Pi }$. To prove the claim, consider such a pair $(\alpha ',\beta ')\in \widehat{\Pi }\setminus \{(\alpha ,\beta )\}$. Let $v \in \mathcal {D}$ be the direction pointing from $(\alpha ',\beta ')$ to $(\alpha ,\beta )$. By applying Proposition 22 repeatedly, we get $|\bar{\pi }_B((\alpha ',\beta ') + v)| \ge 2$. Moreover, as $(\alpha ,\beta )$ is a border pair, we have that $(\alpha ,\beta ) + v$ lies in $\widehat{\Pi }$, and hence so does $(\alpha ',\beta ') + 2v$. It then follows from Lemma 23 that $|\bar{\pi }_A( (\alpha ', \beta ') +v)|= d$, and hence $\left( \bar{\pi }_A ((\alpha ', \beta ')+v) + \bar{\pi }_B ((\alpha ', \beta ')+v) \right) \cap R \ne \emptyset$ by Lemma 20.

It remains to consider the case in which no border pair contains multiple feasible residues. Then there must be a vertex pair with multiple feasible residues. We will show, that there are at most two vertex pairs with $|\pi (\alpha ,\beta )| = 1$. Then these vertices define a tight constraint, that contains all possible hidden solutions. This we can reduce the pattern.

**Fig. 4:**
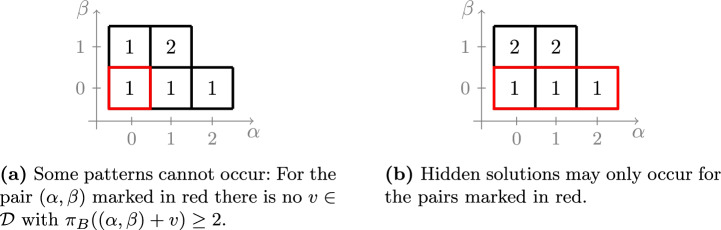
The two cases arising in the proof of Lemma 28. Every square corresponds to a pair $(\alpha ,\beta )\in \widehat{\Pi }$, and the numbers inside indicate the value of $|\bar{\pi }_B(\alpha ,\beta )|$

- Case 2: For all border pairs $(\alpha ,\beta )\in \widehat{\Pi }$, we have $|\bar{\pi }_B(\alpha ,\beta )| = 1$. Since $\widehat{\Pi }$ is a (III) pattern, it has no interior pairs, and there must exist a vertex pair $(\alpha ', \beta ') \in \widehat{\Pi }$ with $|\bar{\pi }_B(\alpha ', \beta ')|\ge 2$. We now distinguish two subcases on the number of vertices of the pattern with a unique feasible residue.
- Case 2.1: There are at least three vertex pairs with $|\bar{\pi }_B (\alpha ,\beta )| =1$.
  By Lemma 27, there are at most four vertex pairs. Hence there is exactly one pair $(\alpha ', \beta ') \in \widehat{\Pi }$ with $|\bar{\pi }_B(\alpha ', \beta ')| \ge 2$, and it is a vertex pair. Moreover, there are four other pairs with a unique feasible residue (three vertex pairs and at least one border pair). By Theorem 26 (i), for each of these pairs $(\alpha ,\beta )\in \widehat{\Pi }$, there exists $v\in \mathcal {D}$ such that $|\bar{\pi }_B((\alpha ,\beta )+ v)| \ge 2$. As $(\alpha ',\beta ')$ is the only pair with $|\bar{\pi }_B(\alpha ',\beta ')|\ge 2$, this implies that there are four directions $d\in \mathcal {D}$ such that $(\alpha ',\beta ')+d\in \widehat{\Pi }$, which contradicts the assumption that $(\alpha ',\beta ')$ is a vertex pair. (Also see Fig. 4.)
- Case 2.2: There are at most two vertex pairs with $|\bar{\pi }_B(\alpha ,\beta )| =1$.
  Consider a border pair $(\alpha ', \beta ')$ and $v\in \mathcal {D}$ such that $(\alpha ', \beta ')\pm v \in \widehat{\Pi }$. The unique constraint of $\widehat{\Pi }$ that is tight at $(\alpha ', \beta ')$ contains two vertex pairs. Both of them must satisfy $|\bar{\pi }_B(\alpha ,\beta )|=1$, for otherwise, Proposition 22 would imply $|\bar{\pi }_B(\alpha ', \beta ')| \ge 2$, contradicting the assumption of Case 2. Additionally, since we assumed to have at most two vertex pairs with $|\bar{\pi }_B(\alpha ,\beta )| =1$, it follows that any vertex pair $(\alpha ,\beta )$ outside this tight constraint must satisfy $|\bar{\pi }_B(\alpha ,\beta )| \ge 2$. Hence, there cannot be a border pair outside this tight constraint. Indeed, assume that there was such a border pair $(\alpha ',\beta ')$ that lies on a different tight constraint. Such tight constraint would have to contain a vertex pair with at least two residues, and using the same argument as above (i.e., Proposition 22), we would get $|\pi _B(\alpha ',\beta ')|\ge 2$, contradicting the assumption of Case 2. Consequently, all pairs $(\alpha ,\beta )\in \widehat{\Pi }$ with $|\bar{\pi }_B (\alpha ,\beta )| = 1$ (those are the only ones where there might be a hidden solution) satisfy the constraint that is tight at $(\alpha ',\beta ')$ with equality. Thus, we can let $\Pi '$ be the pattern shape defined by all $(\alpha ,\beta )$ that satisfy such tight constraint with equality (see Fig. 4b for an example). $\square$

#### Pattern structure of (IV): $\widehat{\Pi }$ has only vertex pairs

For (IV) pattern structure, we first observe that, by Observation 21, if there are any solutions for pairs $(\alpha ,\beta )\in \widehat{\Pi }$ with $|\pi _B(\alpha ,\beta )|\ge 2$, we can also find one efficiently by combining solutions computed for the *A*- and *B*-subproblem when determining $\bar{\pi }_A$ and $\bar{\pi }_B$. The other case, i.e., when no solutions exist for such $(\alpha ,\beta )$, is covered by Theorem 26 (ii). Again, that statement allows to reduce the problem to a new GCTUF problem with the same group *G* and at the same depth *d*, but at least one variable less. Therefore, it allows us to make progress with respect to the number of variables.

### Summary

The above discussion can be summarized in the following theorem. Recall that the depth of an $R$-GCTUF problem is defined as $d:==|G|-|R|$.

#### Theorem 29

Let *G* be a finite abelian group. Consider an $R$-GCTUF problem $\mathcal {P}$ with *n* variables, group *G*, set of target residues *R*, depth $d\le 3$, and a constraint matrix *T* that is a 3-sum of two matrices with $n_A$ and $n_B$ many columns, respectively, such that $n=n_A+n_B$ and $n_A,n_B\ge 2$. Let $p :==\min \{n_A, n_B\}$. Assume furthermore that there is no non-trivial subgroup *H* of *G* with $R = R + H$. Then, after solving at most $(d+1)^3$ many $R$-GCTUF problems with *p* variables, group *G* and depth at most *d*, as well as at most $d(d+1)^2$ $R$-GCTUF problems with $n-p$ variables, group *G* and depth at most $d-1$, one can either

- Find a solution of $\mathcal {P}$ in strongly polynomial time, or
- Determine a single $R$-GCTUF problem $\mathcal {P}'$ with at most $n-p+1$ variables, group *G* and depth *d*, such that $\mathcal {P}$ is feasible if and only if $\mathcal {P}'$ is feasible. Additionally, a solution of $\mathcal {P}'$ can be transformed into a solution of $\mathcal {P}$ in strongly polynomial time.

Furthermore, all involved $R$-GCTUF problems can be constructed in strongly polynomial time.

### Proof of Theorem 16

Consider an $R$-GCTUF problem with group *G*, *n* variables, and depth $d = |G|-|R|\le 3$. If $d=0$, then the problem is an unconstrained TU problem, and thus it is enough to find a vertex solution of the linear relaxation. This can be done in strongly polynomial time using the algorithm of [33]. If $d>0$, we apply Theorem 15 to the constraint matrix *T*. If *T* is covered by one of Items (i) and (ii), then it is a base block matrix itself, so a single call to the oracle for solving a base block problem suffices. Else, *T* is covered by Item (iii) of Theorem 15, and we may assume that *T* decomposes into a 3-sum of two matrices with at least two columns each (see Theorem 15 for the case where a pivot step is necessary). In particular, note that in this case, we have $n\ge 4$. Using Lemma 17 or Theorem 29, we now reduce the problem to one or more smaller problems, until we eventually obtain base block problems, which we solve by an oracle call.

We bound the number of oracle calls triggered by our procedure. Let *f*(*n*, *d*) be the smallest upper bound on the number of oracle calls when starting from an instance with *n* variables and depth at most *d*. We claim that

$$ f(n,d) \le \left( d+1\right) ^{3d}n^{d+3\log _2 (d+1) + 2}\hspace{5.0pt}. $$

To prove this bound, we use induction on $n+d$. First observe that, by the above discussion, $f(n,0)=0$ for any $n\ge 1$, and $f(n,d)=1$ for $n\le 3$ and $d>0$ (in the latter case, we cannot attain Item (iii) of Theorem 15). Now consider an $R$-GCTUF problem with *n* variables and depth *d*. If $R=R+H$ for a non-trivial subgroup *H* of *G*, Lemma 17 allows for a reduction to a single $R$-GCTUF problem at smaller depth. Thus, we end up with at most $f(n,d')$ many base block problems for some $d'<d$. In this case, the induction hypothesis implies the claimed bound on *f*(*n*, *d*) because it is monotone in *d*. In the other case, we apply Theorem 29. Thus, there is a $p \in \{2, \dots , \left\lfloor n/2\right\rfloor \}$ such that the number of base block problems we have to solve is bounded by

$$\begin{aligned}\begin{array}{c} (d+1)^3 f(p,d) + d(d+1)^2 f(n-p,d-1) + f(n-p+1,d)\\ \le \left( d+1\right) ^{3d}n^{d+3\log _2 (d+1) + 2} \underbrace{\left( \left( \frac{p}{n}\right) ^2 + \frac{n-p}{n^2} + \left( \frac{n-p+1}{n}\right) ^2 \right) }_{\le 1}\\ \le \left( d+1\right) ^{3d}n^{d+3\log _2 (d+1) + 2}\hspace{5.0pt}, \end{array}\end{aligned}$$

$$\begin{aligned}&(d+1)^3 f(p,d) + d(d+1)^2 f(n-p,d-1) + f(n-p+1,d)\\&\le \left( d+1\right) ^{3d}n^{d+3\log _2 (d+1) + 2} \underbrace{\left( \left( \frac{p}{n}\right) ^2 + \frac{n-p}{n^2} + \left( \frac{n-p+1}{n}\right) ^2 \right) }_{\le 1}\\&\le \left( d+1\right) ^{3d}n^{d+3\log _2 (d+1) + 2}\hspace{5.0pt}, \end{aligned}$$

proving the claimed bound on *f*(*n*, *d*). Here, we use that

$$\begin{aligned} (d+1)^3 f(p,d)&\le \left( d+1\right) ^{3d+3}p^{d+3\log _2 (d+1) + 2}\\&= \underbrace{(d+1)^3 \left( \frac{p}{n}\right) ^{d+3\log _2 (d+1)}}_{\le 1}\left( d+1\right) ^{3d}n^{d+3\log _2 (d+1) + 2} \left( \frac{p}{n}\right) ^{2} \\&\le \left( d+1\right) ^{3d}n^{d+3\log _2 (d+1) + 2}\left( \frac{p}{n}\right) ^2\hspace{5.0pt}, \end{aligned}$$

as $p/n\le 1/2$ and hence $\left( p/n\right) ^{d+3\log _2 (d+1)} \le (d+1)^{-3}$, together with

$$\begin{aligned}&d(d+1)^2 f(n-p,d-1) \\&\le d(d+1)^2 d^{3d-3}(n-p)^{d-1+3\log _2 d + 2}\\&\le \underbrace{\frac{d(d+1)^2}{(d+1)^3} \left( \frac{n-p}{n}\right) ^{d+3\log _2 (d+1)}}_{\le 1}\left( d+1\right) ^{3d}n^{d+3\log _2 (d+1) + 2} \left( \frac{n-p}{n^{2}}\right) \\&\le \left( d+1\right) ^{3d}n^{d+3\log _2 (d+1) + 2} \left( \frac{n-p}{n^{2}}\right) \hspace{5.0pt}. \end{aligned}$$

$$\square $$

## Data Availability

Not applicable.

## Appendix A Adapted proofs of base block results

In this section, we discuss how to extend the proofs of [3, Section 4] for congruency-constrained TU problems with a base block constraint matrix, i.e., matrices covered by Items (i) and (ii) of Theorem 15. Note that the case of the constraint matrix being the transposed of a network matrix has been settled in Sect. 4, so we will focus on the constraint matrix being a network matrix or being obtainable from a constant size matrix. The congruency constraints that were used previously may equivalently be formulated as constraints in a cyclic group $\mathbb {Z}/m\mathbb {Z}$, and it turns out that all arguments extend to general finite abelian groups, i.e., the setting that we need for our purposes. In this appendix, we recall the proofs of [3] and comment on the mostly straightforward modifications for the sake of completeness.

To this end, let us first define *group-constrained TU problems* (GCTU problems) to be the optimization variant of GCTUF problems, i.e., where additionally to the GCTUF setup, we are given an objective $c\in \mathbb {R}^n$ that we want to minimize over all feasible solutions of the GCTUF problem. Note that we can always assume to start with a GCTU problem whose relaxation (i.e., the problem obtained after dropping the group constraint) is feasible, which we can check in strongly polynomial time; for otherwise, the GCTU problem is clearly infeasible. Hence, we assume feasibility of the relaxation throughout this section. To start with, let us recall the definition of a *network matrix*.

### Definition 30

A matrix *T* is a *network matrix* if the rows of *T* can be indexed by the edges of a directed spanning tree (*V*, *U*), and the columns can be indexed by the edges of a directed graph (*V*, *A*) on the same vertex set, such that for every arc $a=(v, w)\in A$ and every arc $u\in U$,

$$ T_{u,a} = {\left\{ \begin{array}{ll} 1 &  \text {if the unique } v-w \text { path in } U \text { passes through } u \text { forwardly,}\\ 0 &  \text {if the unique } v-w \text { path in } U \text { does not pass through } u,\\ -1 &  \text {if the unique } v-w \text { path in } U \text { passes through } u \text { backwardly.} \end{array}\right. } $$

In the subsequent two sections, we distinguish the two remaining cases of base block matrices, namely whether the constraint matrix *T* of the GCTU problem that we consider is a network matrix or a matrix stemming from the constant-size matrices given in Item (ii) of Theorem 15.

### A.1 Network matrix base block GCTU problems

In this section, we discuss the extension from congruency constraints to group constraints in the case where the constraint matrix is a network matrix. Concretely, this will provide the following result.

#### Theorem 31

Let *G* be a finite abelian group. There is a strongly polynomial time randomized algorithm for GCTU problems with group *G*, unary encoded objectives, and constraint matrices that are network matrices.

In this case, one can exploit the graph structure that comes with network matrices to interpret GCTU problems with network constraint matrices as minimum-cost group-constrained circulation problems in certain directed graphs. To get started, let us recall that a circulation *f* in a directed graph $H=(V,A)$ with capacities $u:A\rightarrow \mathbb {Z}_{\ge 0}$ is a mapping $f:A\rightarrow \mathbb {Z}_{\ge 0}$ such that $f(a)\le u(a)$ for every arc $a\in A$, and $f(\delta ^+(v)) = f(\delta ^-(v))$ for every vertex $v\in V$. Given arc lengths $\ell :A\rightarrow \mathbb {Z}$, the length of a circulation *f* is $\ell (f):==\sum _{a\in A}\ell (a)f(a)$. Note that here, arc lengths are allowed to be negative. A group-constrained circulation problem is formally defined as follows.

In [3, Lemma 4.2], a reduction from GCTU problems with a network matrix as constraint matrix to GCC is presented for the case of a cyclic group $G=\mathbb {Z}/m\mathbb {Z}$. In this reduction, the only property of $\mathbb {Z}/m\mathbb {Z}$ that is exploited is [3, Lemma 2.2]. Thus, replacing this statement by its group-constrained analogue Lemma 19 proved earlier immediately gives the following.

#### Lemma 32

GCTU problems with group *G*, objective vector *c*, and constraint matrices that are network matrices can be reduced in strongly polynomial time to GCC problems with group *G*, capacities *u* within $\{0, \ldots , |G|-1\}$, and arc lengths $\ell$ with $\Vert \ell \Vert _\infty \le \Vert c\Vert _{\infty }$.

Now, one can finish the proof of Theorem 31 by exploiting a connection to the so-called *exact length circulation problem*, where the goal is to find a circulation whose length is equal to a given value. The reduction follows the one given in [3, Lemma 4.3], but needs to be slightly adapted in order to capture group constraints.  Exact length circulation problems can be solved using a randomized pseudopolynomial algorithm, as shown by [17]. They reduce the problem to an exact cost perfect matching problem, which can then be reduced to computing the coefficients of a well-defined polynomial. The following theorem summarizes the result of [17] for XLC .

#### Theorem 33

([17]) There is a randomized algorithm for XLC problems in a directed graph $H=(V,A)$ with capacities $u:A\rightarrow \mathbb {Z}_{\ge 0}$ in time $\textrm{poly}(|V|, \max _{a\in A}u(a), \max _{a\in A}|\ell (a)|)$.

Thus, it remains to extend the connection between GCC and XLC problems from the congruency-constrained to the group-constrained setting. To this end, we recall the reduction for a single congruency constraint modulo *m*: Taking an integer representative in $\{0, \dots , m-1\}$ for each residue modulo *m*, we may construct new arc lengths that capture the residue representative *r*(*a*) and original arc lengths $\ell (a)$ independently (e.g., by using $\tilde{\ell }(a) = c\cdot \ell (a) + r(a)$ for a large enough constant *c*). Guessing both the length and the residue class of an optimal solution of the GCC problem is then equivalent to guessing a target *L* with respect to the new lengths, hence the reduction can be achieved by binary search.

For group constraints, we recall that we may equivalently interpret a group constraint as multiple congruency constraints, which can then be integrated into new arc lengths $\tilde{\ell }$ at different orders of magnitude. Formally, this leads to the following lemma, which is an analogue of [3, Lemma 4.3].

#### Lemma 34

A GCC problem in a graph $H=(V,A)$ with group *G*, arc lengths $\ell :A\rightarrow \mathbb {Z}$, and capacities $u:A\rightarrow \{0, 1, \ldots , |H|-1\}$ can be polynomially reduced to $\textrm{poly}(|G|, |V|, |A|, \max _{a\in A}|\ell (a)|)$ many XLC problems in *G* with the same capacities.

#### Proof

First, we may decompose $G\cong \mathbb {Z}/m_1\mathbb {Z} \times \cdots \times \mathbb {Z}/m_k\mathbb {Z}$ for some $k\in \mathbb {Z}_{\ge 1}$. For every $i\in [k]$, let $\varphi _i :G \rightarrow \mathbb {Z}/m_i\mathbb {Z} \rightarrow \mathbb {Z}$ denote the corresponding natural projection maps composed with the natural mapping of a residue in $\mathbb {Z}/m_i\mathbb {Z}$ to a representative in $\{0, \dots , m_i-1\}$. Set $m:==|G|$.

Define $\tilde{\ell }:A\rightarrow \mathbb {Z}$ for every arc $a\in A$ as

$$\tilde{\ell }(a)=\ell (a)\cdot m^{2k}|A|^k + \sum _{i=1}^k m^{2i-2}|A|^{i-1}\varphi _i(\eta (a))\hspace{5.0pt}.$$

We thus have $\tilde{\ell }(f) = \ell (f)\cdot m^{2k}|A|^k + \sum _{i=1}^k m^{2i-2}|A|^{i-1}\varphi _i(\eta (f))$. Observe that $\sum _{a\in A}\varphi _i(\eta (a))f(a) < m^2 |A|$ for every $1\le i\le k$, hence from $\tilde{\ell }(f)$, one can retrieve $\ell (f)$ as well as $\sum _{a\in A}\varphi _i(\eta (a))f(a)$ for every $i\in [k]$. Consequently, finding a circulation of length *L* with $\sum _{a\in A}\eta (a)f(a)= r$ is equivalent to solving XLC problems in *H* with respect to lengths $\tilde{\ell }$ and with target length $\tilde{L}=L\cdot m^{2k}|A|^k + \sum _{i=1}^km^{2i-2}|A|^{i-1} (d_im_i + \varphi _i(r))$ for all tuples $(d_1, \dots , d_k)\in \{0,\ldots , m|A|-1\}^k$. We can find the smallest *L* for which there is a GCC solution of length *L* by binary search in $O(k\log (m|A|\cdot \max _{a\in A}|\ell (a)|))$ iterations, because $|\ell (f)| = \left| \sum _{a\in A}\ell (a)f(a)\right| \le m|A|\cdot \max _{a\in A}|\ell (a)|$. Altogether, this gives the desired result. $\square$

Combining Lemmas 32 and 34 and 33 readily implies Theorem 31.

### A.2 Matrices stemming from particular constant-size matrices

To complete the discussion of base block GCTU problems, we now cover GCTU problems with constraint matrices covered by Item (ii) of Theorem 15. In other words, these are matrices *T* that can be obtained from the two matrices

A1 $$\begin{aligned} \begin{pmatrix} 1 &  -1 &  0 &  0 &  -1 \\ -1 &  1 &  -1 &  0 &  0 \\ 0 &  -1 &  1 &  -1 &  0 \\ 0 &  0 &  -1 &  1 &  -1 \\ -1 &  0 &  0 &  -1 &  1 \end{pmatrix} \quad \text {and}\quad \begin{pmatrix} 1 &  1 &  1 &  1 &  1 \\ 1 &  1 &  1 &  0 &  0 \\ 1 &  0 &  1 &  1 &  0 \\ 1 &  0 &  0 &  1 &  1 \\ 1 &  1 &  0 &  0 &  1 \end{pmatrix} \end{aligned}$$

by repeatedly appending unit vector rows or columns, appending a copy of a row or column, and inverting the sign of a row or column.

To tackle problems with such constraint matrices, [3, Section 4.3] more generally considers constraint matrices *T* that are obtained from a *core* matrix *C* through the above operations. (Concretely, in the case relevant here, *C* will be one of the two matrices in (A1).) It is shown that if *C* has $\ell$ columns, then there are integral vectors $s_1,\ldots ,s_\ell$ such that once the values $s_i^\top x$ are known for all $i\in [\ell ]$, the inequality system $Tx\le b$ can be reduced to an equivalent system $T'x\le b'$ on the same variables *x*, where $T'$ is a network matrix and the transpose of a network matrix at the same time, and $b'$ is integral. By exploiting a proximity statement for congruency-constrained TU problems, [3] observe that it suffices to consider values $s_i^\top x\in \{-m+1,\ldots ,m-1\}$, where *m* is the modulus of the congruency-constraint, and they conclude that there are only $(2m-1)^\ell$ possible combinations of values for $s_i^\top x$ to be enumerated, which can be done in polynomial time for constant $\ell$. To extend this reasoning from congruency constraints (i.e., constraints in a cyclic group $G=\mathbb {Z}/m\mathbb {Z}$) to constraints in general finite abelian groups, we observe that the aforementioned proximity statement relies on a decomposition theorem for solutions of totally unimodular systems [3, Lemma 2.1] and the property of cyclic groups that we generalized in Lemma 19. Thereby, it is again a matter of replacing modular arithmetic by calculations in a general finite abelian group *G* to obtain the following result.

#### Lemma 35

Let *G* be a finite abelian group and consider a GCTU problem with group *G* and a constraint matrix *T* that can be obtained from a matrix *C* with $\ell$ columns by repeatedly appending unit vector rows or columns, appending a copy of a row or column, and inverting the sign of a row or column. Then, the GCTU problem can be reduced to $(2|G|-1)^\ell$ many GCTU problems with group *G* and constraint matrices of size linear in the size of *T* that are network matrices and transposes of network matrices at the same time.

For feasibility problems, we may thus exploit Theorem 7 to obtain the following direct corollary.

#### Corollary 36

Let *G* be a finite abelian group. There is a strongly polynomial time algorithm for solving GCTUF problems with group *G* and a constraint matrix covered by Item (ii) of Theorem 15.

## Footnotes

1 A weaker variant of the conjecture claims efficient solvability of IPs with *totally* $\Delta$-*modular* constraint matrices, where all subdeterminants are bounded by $\Delta$ in absolute value. The conjecture involving $\Delta$-modular matrices implies the weaker variant. Indeed, an IP $\min \{c^\top x :Ax \le b, x\in \mathbb {Z}^n\}$ with a totally $\Delta$-modular constraint matrix can be reformulated as $\min \{c^\top (x^+-x^-) :A(x^+-x^-)\le b, x^+, x^- \in \mathbb {Z}_{\ge 0}^n\}$. It is not hard to see that the constraint matrix of the new LP remains totally $\Delta$-modular; moreover, it has full column rank because of the non-negativity constraints.

2 To capture an MCCTU : problem as a strictly $\Delta$-modular IP, replace each congruency constraint $\gamma _i^\top x \equiv r_i\,(\text{ mod } m_i)$ by an equality constraint $\gamma _i^\top x + m_i y_i = r$ with $y_i\in \mathbb {Z}$. The corresponding constraint matrix is strictly $\Delta$-modular for $\Delta =\prod _{i=1}^q m_i$.

3 Note that we might have $\widetilde{m}_i=0$ for some $i\in [n]$. In this case, $\gcd (\Delta , \widetilde{m}_i)=\Delta$ and hence $m_i=1$, so the corresponding congruency constraint is always satisfied.

4 In fact, the proof in [3] claims a reduction to a submodular minimization problem, but shows the stronger one presented here.

5 We recall that a *lattice* $\mathcal {L}\subseteq 2^N$ is a set family such that for any $A,B\in \mathcal {L}$, we have $A\cap B, A\cup B\in \mathcal {L}$. We assume such a lattice to be given by a compact encoding in a directed acyclic graph *H* on the vertex set *N* such that $X\subseteq N$ is an element of the lattice if and only if $\delta _H^-(X)=\emptyset$ (cf. [36, Section10.3]). Here, as usual, in a digraph $G=(V,A)$ and for $X\subseteq V$, we denote by $\delta ^+(X)$ and $\delta ^-(X)$ the arcs in *A* leaving and entering *X*, respectively. Moreover, we write $\delta ^\pm (v):==\delta ^\pm (\{v\})$ for $v\in V$.

## References

[1] Artmann, S., Weismantel, R., Zenklusen, R.: A strongly polynomial algorithm for bimodular integer linear programming. In: Proceedings of the 49th Annual ACM Symposium on Theory of Computing (STOC ’17), 1206–1219 (2017). 10.1145/3055399.3055473

[2] Fiorini, S., Joret, G., Weltge, S., Yuditsky, Y.: Integer programs with bounded subdeterminants and two nonzeros per row. In: Proceedings of the 62nd Annual Symposium on Foundations of Computer Science (FOCS ’22), pp. 13–24 (2022). 10.1109/FOCS52979.2021.00011

[3] Nägele, M., Santiago, R., Zenklusen, R.: Congruency-constrained TU problems beyond the bimodular case. In: Proceedings of the 33rd Annual ACM-SIAM Symposium on Discrete Algorithms (SODA ’22), pp. 2743–2790 (2022). 10.1137/1.9781611977073.108

[4] Padberg, M.W., Rao, M.R.: Odd minimum cut-sets and -matchings. Math. Oper. Res. **7**(1), 67–80 (1982). 10.1287/moor.7.1.67

[5] Barahona, F., Conforti, M.: A construction for binary matroids. Discret. Math. **66**(3), 213–218 (1987). 10.1016/0012-365X(87)90097-5

[6] Grötschel, M., Lovász, L., Schrijver, A.: Corrigendum to our paper ‘The ellipsoid method and its consequences in combinatorial optimization’. Combinatorica **4**(4), 291–295 (1984). 10.1007/BF02579139

[7] Goemans, M.X., Ramakrishnan, V.S.: Minimizing submodular functions over families of sets. Combinatorica **15**(4), 499–513 (1995). 10.1007/BF01192523

[8] Nägele, M., Sudakov, B., Zenklusen, R.: Submodular minimization under congruency constraints. Combinatorica **39**(6), 1351–1386 (2019). 10.1007/s00493-019-3900-1

[9] Nägele, M., Zenklusen, R.: A new contraction technique with applications to congruency-constrained cuts. Math. Program. **183**, 455–481 (2020). 10.1007/s10107-020-01498-x

[10] Veselov, S.I., Chirkov, A.J.: Integer program with bimodular matrix. Discret. Optim. **6**(2), 220–222 (2009). 10.1016/j.disopt.2008.12.002

[11] Gribanov, D., Shumilov, I., Malyshev, D., Pardalos, P.: On -modular integer linear problems in the canonical form and equivalent problems. J. Global Optim. (2022). 10.1007/s10898-022-01165-9

[12] Barahona, F., Pulleyblank, W.R.: Exact arborescences, matchings and cycles. Discrete Appl. Math. **16**(2), 91–99 (1987). 10.1016/0166-218X(87)90067-9

[13] Galluccio, A., Loebl, M.: On the theory of Pfaffian orientations. I. Perfect matchings and permanents. The Electronic Journal of Combinatorics, 6–6 (1999)

[14] Seymour, P.D.: Decomposition of regular matroids. J. Combin. Theory Series B **28**(3), 305–359 (1980). 10.1016/0095-8956(80)90075-1

[15] Dinitz, M., Kortsarz, G.: Matroid secretary for regular and decomposable matroids. SIAM J. Comput. **43**(5), 1807–1830 (2014). 10.1137/13094030X

[16] Aprile, M., Fiorini, S.: Regular matroids have polynomial extension complexity. Math. Oper. Res. **47**(1), 540–559 (2021). 10.1287/moor.2021.1137

[17] Camerini, P.M., Galbiati, G., Maffioli, F.: Random pseudo-polynomial algorithms for exact matroid problems. J. Algorithms **13**, 258–273 (1992). 10.1016/0196-6774(92)90018-8

[18] Di Summa, M., Eisenbrand, F., Faenza, Y., Moldenhauer, C.: On largest volume simplices and sub-determinants. In: Proceedings of the 26th Annual ACM-SIAM Symposium on Discrete Algorithms (SODA ’15), pp. 315–323 (2015). 10.1137/1.9781611973730.23

[19] Nikolov, A.: Randomized rounding for the largest simplex problem. In: Proceedings of the 47th Annual ACM Symposium on Theory of Computing (STOC ’15), pp. 861–870 (2015). 10.1145/2746539.2746628

[20] Artmann, S., Eisenbrand, F., Glanzer, C., Oertel, T., Vempala, S., Weismantel, R.: A note on non-degenerate integer programs with small sub-determinants. Oper. Res. Lett. **44**(5), 635–639 (2016). 10.1016/j.orl.2016.07.004

[21] Glanzer, C., Stallknecht, I., Weismantel, R.: On the recognition of -modular matrices. In: Proceedings of the 22nd International Conference on Integer Programming and Combinatorial Optimization (IPCO ’21), pp. 238–251 (2021). 10.1007/978-3-030-73879-2_17

[22] Heller, I.: On linear systems with integral valued solutions. Pac. J. Math. **7**(3), 1351–1364 (1957). 10.2140/pjm.1957.7.1351

[23] Glanzer, C., Weismantel, R., Zenklusen, R.: On the number of distinct rows of a matrix with bounded subdeterminants. SIAM J. Discret. Math. **32**(3), 1706–1720 (2018). 10.1137/17M1125728

[24] Lee, J., Paat, J., Stallknecht, I., Xu, L.: Polynomial upper bounds on the number of differing columns of -modular integer programs. Math. Oper. Res. (2022). 10.1287/moor.2022.1339

[25] Averkov, G., Schymura, M.: On the maximal number of columns of a -modular matrix. In: Proceedings of the 23rd International Conference on Integer Programming and Combinatorial Optimization (IPCO ’22), pp. 29–42 (2022). 10.1007/978-3-031-06901-7_3

[26] Bonifas, N., Di Summa, M., Eisenbrand, F., Haehnle, N., Niemeier, M.: On sub-determinants and the diameter of polyhedra. Discrete Comput. Geometry **52**(1), 14–102115 (2014). 10.1007/s00454-014-9601-x

[27] Eisenbrand, F., Vempala, S.: Geometric random edge. Math. Program. **164**(1), 325–339 (2017). 10.1007/s10107-016-1089-0

[28] Gribanov, D.V., Veselov, S.I.: On integer programming with bounded determinants. Optim. Lett. **10**(6), 1169–1177 (2016). 10.1007/s11590-015-0943-y

[29] Gribanov, D.V., Zolotykh, N.Y.: On lattice point counting in -modular polyhedra. Optim. Lett. **16**, 1991–2018 (2021). 10.1007/s11590-021-01744-x

[30] Gribanov, D.V.: An FPTAS for the -modular multidimensional knapsack problem. In: Proceedings of the International Conference on Mathematical Optimization Theory and Operations Research (MOTOR), pp. 79–95 (2021). 10.1007/978-3-030-77876-7_6

[31] Lee, J., Paat, J., Stallknecht, I., Xu, L.: Improving proximity bounds using sparsity. In: Proceedings of the 6th International Symposium on Combinatorial Optimization (ISCO ’20), pp. 115–127 (2020). 10.1007/978-3-030-53262-8_10

[32] Paat, J., Schlöter, M., Weismantel, R.: The integrality number of an integer program. Math. Program. **192**, 271–291 (2022). 10.1007/s10107-021-01651-0

[33] Tardos, É.: A strongly polynomial algorithm to solve combinatorial linear programs. Oper. Res. **34**(2), 250–256 (1986). 10.1287/opre.34.2.250

[34] Kannan, R., Bachem, A.: Polynomial algorithms for computing the smith and hermite normal forms of an integer matrix. SIAM J. Comput. **8**, 499–507 (1979). 10.1137/0208040

[35] Schrijver, A.: Theory of Linear and Integer Programming. John Wiley & Sons

[36] Grötschel, M., Lovász, L., Schrijver, A.: Geometric Algorithms and Combinatorial Optimization. Algorith. Combin. (1993). 10.1007/978-3-642-78240-4

[37] Artmann, S.: Optimization of bimodular integer programs and feasibility for three-modular base block ips. PhD thesis, ETH Zurich (2020). 10.3929/ethz-b-000420070

